# Supplementary material for: Estimating Temporally Variable Selection Intensity from Ancient DNA Data
Source: Mol Biol Evol. 2023 Jan 20;40(3):msad008. doi: 10.1093/molbev/msad008 (PMC10063216; doi:10.1093/molbev/msad008)
Supplement: msad008_Supplementary_Data [file msad008_supplementary_data.zip › ZH2021_Supplementary_Material.pdf]

Scenario	Sel. coeff. s^-		Sel. coeff. s^+	
	Bias	RMSE	Bias	RMSE
A	0.00126	0.00391	0.00684	0.00952
B	0.00124	0.00424	0.00229	0.00708
C	0.00054	0.00399	0.00378	0.00806
D	0.00022	0.00348	0.00073	0.00595
E	0.00081	0.00354	0.00104	0.00603
F	0.00043	0.00402	0.00053	0.00618

(a) Scenario 1: $s^- < 0$ and $s^+ < s^-$.

Scenario	Sel. coeff. s^-		Sel. coeff. s^+	
	Bias	RMSE	Bias	RMSE
A	0.00258	0.00438	0.00358	0.00646
B	0.00111	0.00411	0.00100	0.00507
C	0.00131	0.00387	0.00167	0.00627
D	0.00067	0.00369	−0.00018	0.00592
E	0.00042	0.00372	−0.00005	0.00541
F	0.00056	0.00358	−0.00051	0.00577

(b) Scenario 2: $s^- < 0$ and $s^+ = s^-$.

Scenario	Sel. coeff. s^-		Sel. coeff. s^+	
	Bias	RMSE	Bias	RMSE
A	0.00503	0.00777	−0.00537	0.00896
B	0.00214	0.00585	−0.00263	0.00638
C	0.00244	0.00547	−0.00331	0.00725
D	0.00128	0.00499	−0.00165	0.00593
E	0.00038	0.00462	−0.00104	0.00558
F	0.00052	0.00432	−0.00018	0.00563

(c) Scenario 3: $s^- < 0$ and $s^+ > s^-$.

Table S2: Mean bias and RMSE in MAP estimates of the selection coefficients across different data qualities and selection scenarios, corresponding to Figure 3. Mean bias and RMSE are calculated with 200 replicates for each combination. Data qualities (scenarios A–F) are described in Table 1, and selection scenarios (scenarios 1–9) are described in Table 2.

Scenario	Sel. coeff. s^-		Sel. coeff. s^+	
	Bias	RMSE	Bias	RMSE
A	−0.00023	0.00347	0.00551	0.00834
B	0.00028	0.00348	0.00191	0.00528
C	0.00022	0.00366	0.00265	0.00571
D	0.00064	0.00362	−0.00027	0.00508
E	−0.00001	0.00338	0.00079	0.00432
F	0.00063	0.00368	−0.00035	0.00424

(d) Scenario 4: $s^- = 0$ and $s^+ < s^-$.

Scenario	Sel. coeff. s^-		Sel. coeff. s^+	
	Bias	RMSE	Bias	RMSE
A	0.00020	0.00389	0.00026	0.00300
B	−0.00001	0.00437	0.00044	0.00337
C	0.00015	0.00368	0.00022	0.00292
D	0.00015	0.00422	0.00040	0.00330
E	−0.00003	0.00391	0.00028	0.00301
F	0.00007	0.00380	0.00040	0.00328

(e) Scenario 5: $s^- = 0$ and $s^+ = s^-$.

Scenario	Sel. coeff. s^-		Sel. coeff. s^+	
	Bias	RMSE	Bias	RMSE
A	−0.00031	0.00368	−0.00460	0.00722
B	−0.00084	0.00409	−0.00134	0.00548
C	−0.00054	0.00394	−0.00195	0.00571
D	−0.00061	0.00411	0.00021	0.00548
E	−0.00060	0.00365	−0.00034	0.00494
F	−0.00086	0.00397	0.00037	0.00427

(f) Scenario 6: $s^- = 0$ and $s^+ > s^-$.

Table S2: Mean bias and RMSE in MAP estimates of the selection coefficients across different data qualities and selection scenarios, corresponding to Figure 3, continued.

Scenario	Sel. coeff. s^-		Sel. coeff. s^+	
	Bias	RMSE	Bias	RMSE
A	-0.00558	0.00834	0.00585	0.00951
B	-0.00259	0.00619	0.00200	0.00611
C	-0.00265	0.00580	0.00195	0.00624
D	-0.00128	0.00529	0.00021	0.00593
E	-0.00064	0.00452	0.00002	0.00569
F	-0.00067	0.00475	0.00034	0.00552

(g) Scenario 7: $s^- > 0$ and $s^+ < s^-$.

Scenario	Sel. coeff. s^-		Sel. coeff. s^+	
	Bias	RMSE	Bias	RMSE
A	-0.00295	0.00521	-0.00383	0.00721
B	-0.00131	0.00415	-0.00093	0.00596
C	-0.00104	0.00371	-0.00219	0.00628
D	-0.00066	0.00389	0.00001	0.00547
E	-0.00090	0.00385	-0.00033	0.00598
F	-0.00092	0.00359	0.00079	0.00572

(h) Scenario 8: $s^- > 0$ and $s^+ = s^-$.

Scenario	Sel. coeff. s^-		Sel. coeff. s^+	
	Bias	RMSE	Bias	RMSE
A	-0.00167	0.00412	-0.00713	0.00982
B	-0.00124	0.00434	-0.00294	0.00792
C	-0.00142	0.00380	-0.00284	0.00744
D	-0.00019	0.00350	-0.00006	0.00687
E	-0.00047	0.00332	-0.00082	0.00597
F	-0.00038	0.00365	-0.00068	0.00612

(i) Scenario 9: $s^- > 0$ and $s^+ > s^-$.

Table S2: Mean bias and RMSE in MAP estimates of the selection coefficients across different data qualities and selection scenarios, corresponding to Figure 3, continued.

Sel. coeff. s	Bias	RMSE
$s \in [-0.050, -0.010)$	0.00106	0.00298
$s \in [-0.010, -0.005)$	0.00016	0.00231
$s \in [-0.005, -0.001)$	-0.00026	0.00205
$s \in [-0.001, 0)$	0.00006	0.00206
$s \in \{0\}$	0.00019	0.00201
$s \in (0, 0.001]$	0.00020	0.00218
$s \in (0.001, 0.005]$	-0.00003	0.00200
$s \in (0.005, 0.010]$	-0.00015	0.00231
$s \in (0.010, 0.050]$	-0.00077	0.00310

Table S3: Mean bias and RMSE in MAP estimates of the selection coefficient across different ranges of the selection coefficient s with the parameters $\phi = 0.85$ and $\psi = 1$ (*i.e.*, scenario D in Table 1), corresponding to Figure 4. Mean bias and RMSE are calculated with 200 replicates for each case.

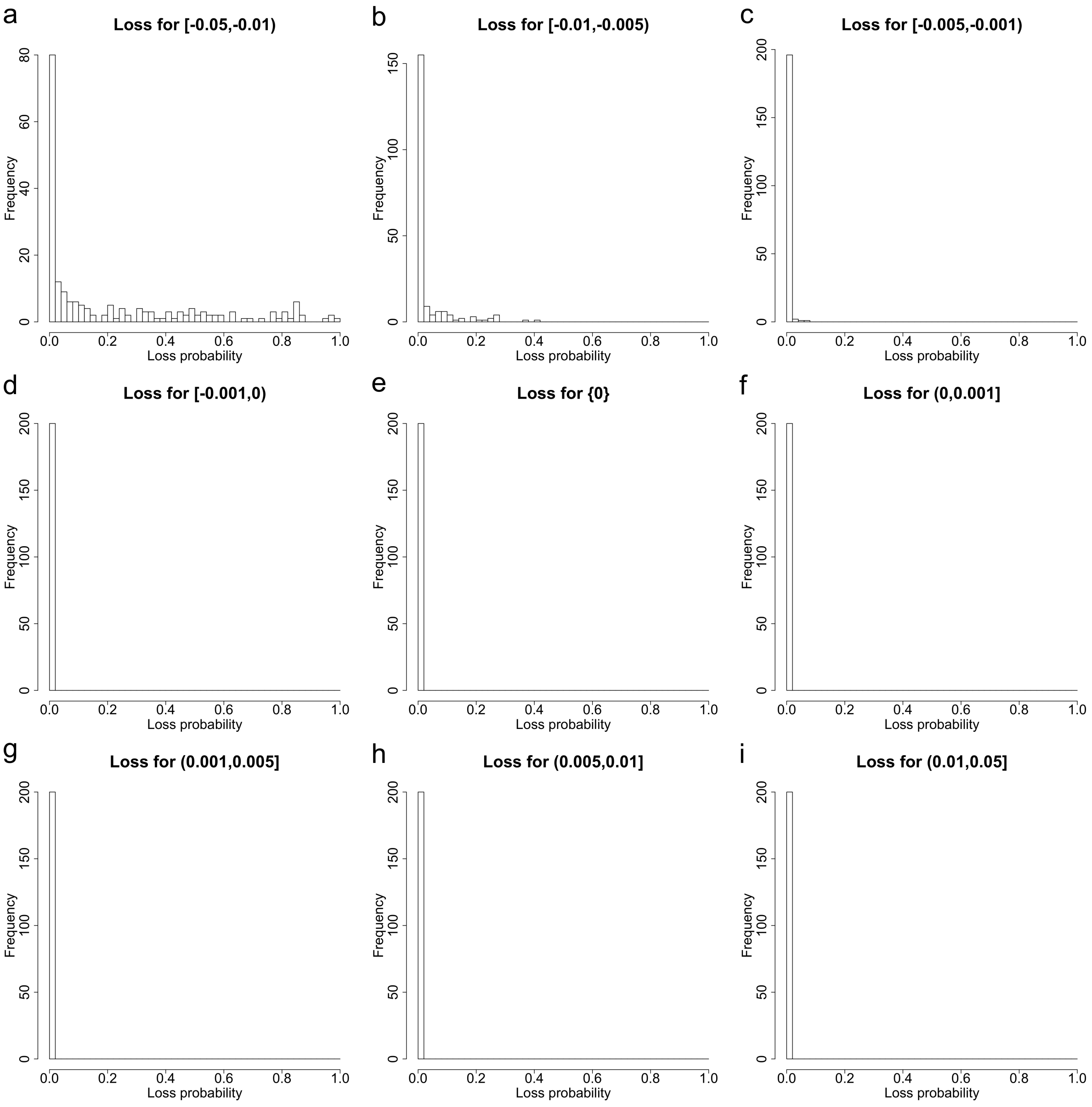

(a) Histograms of loss probabilities.

Figure S1: Empirical distributions for the loss probability and the fixation probability across different ranges of the selection coefficient s with the parameters $\phi = 0.85$ and $\psi = 1$ (*i.e.*, scenario D in Table 1), corresponding to Figure 4.

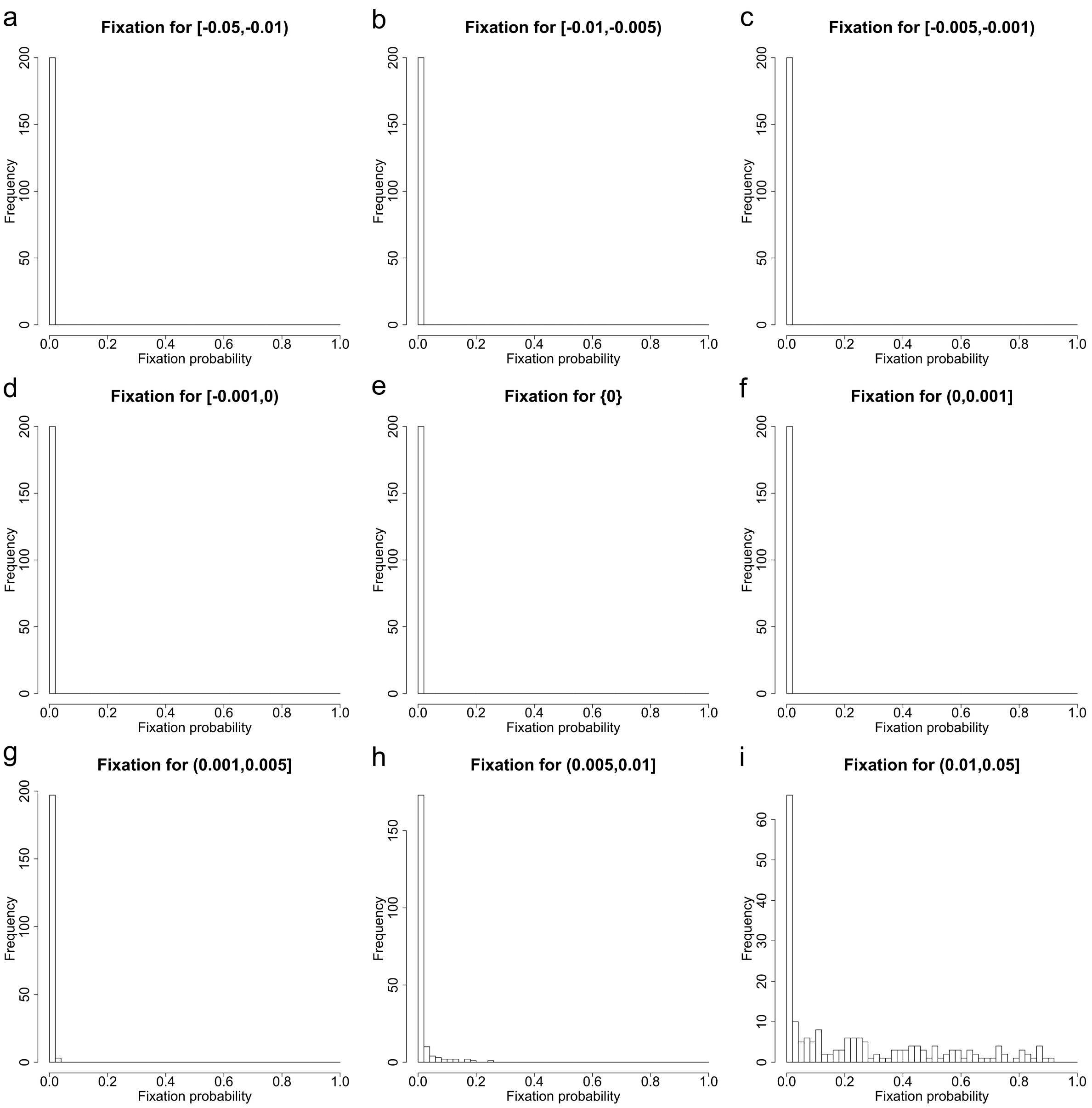

(b) Histograms of fixation probabilities.

Figure S1: Empirical distributions for the loss probability and the fixation probability across different ranges of the selection coefficient s with the parameters $\phi = 0.85$ and $\psi = 1$ (*i.e.*, scenario D in Table 1), corresponding to Figure 4, continued.

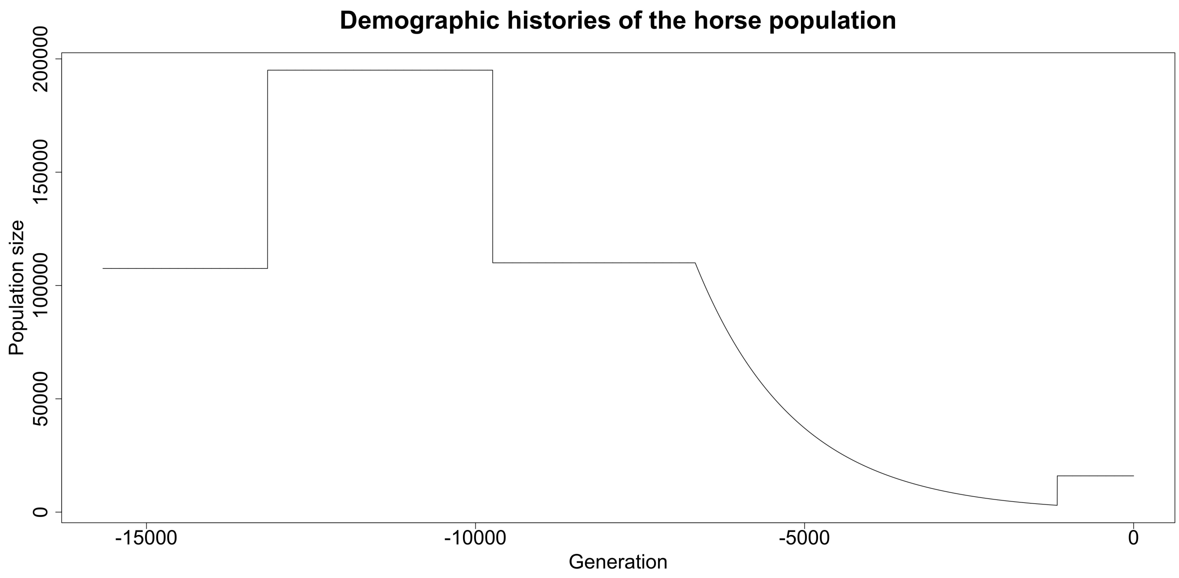

Figure S2: Demographic histories of the horse population reported in Der Sarkissian et al. (2015).

Gene	Event	Sel. coeff.	MAP est.	95% HPD	Prob. for -ve.	Prob. for +ve.
		s^-	0.00178	[−0.00120, 0.00660]	0.0960	0.9040
ASIP	DOM	s^+	0.00032	[−0.00225, 0.00313]	0.4110	0.5890
		Δs	−0.00208	[−0.00708, 0.00314]	0.7790	0.2210
		s^-	−0.03003	[−0.09277, 0.09973]	0.6695	0.3305
MC1R	DOM	s^+	0.01159	[0.00787, 0.01736]	0.0000	1.0000
		Δs	0.04389	[−0.08908, 0.10579]	0.2525	0.7475
		s^-	0.00392	[−0.00097, 0.01031]	0.0650	0.9350
KIT13	EMA	s^+	−0.02836	[−0.06270, 0.00152]	0.9685	0.0315
		Δs	−0.03259	[−0.07003, 0.00078]	0.9690	0.0310
		s^-	−0.00049	[−0.00421, 0.00294]	0.6170	0.3830
TRPM1	DOM	s^+	−0.00775	[−0.01387, −0.00046]	0.9800	0.0200
		Δs	−0.00665	[−0.01419, 0.00250]	0.9420	0.0580

Table S5: MAP estimates of the selection coefficients and their changes with their 95% HPD intervals, as well as posterior probabilities for negative selection/change and positive selection/change, for *ASIP*, *MC1R*, *KIT13* and *TRPM1*, corresponding to Figures 7–10. Prob. for -ve. and +ve. stand for posterior probabilities for negative selection/change and positive selection/change, respectively. DOM and EMA stand for domestication and Early Middle Ages, respectively.

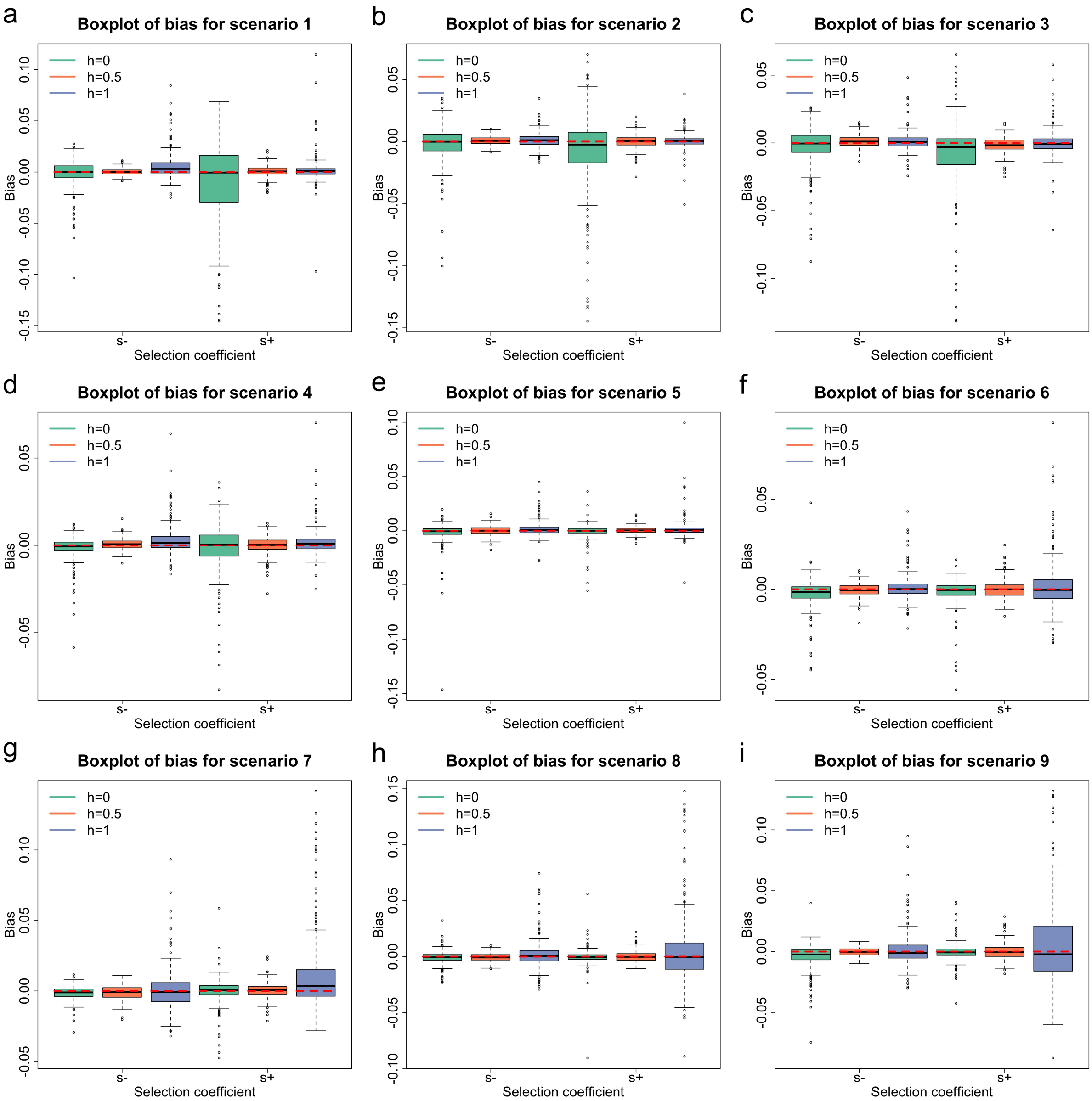

Figure S3: Empirical distributions for the bias in MAP estimates of the selection coefficients across different gene actions and selection scenarios with the parameters $\phi = 0.85$ and $\psi = 1$ (*i.e.*, scenario D in Table 1). For each combination, we repeatedly run our procedure on 200 simulated datasets. Selection scenarios (scenarios 1–9) are described in Table 2. (a)–(i) Boxplots of the bias for scenarios 1–9.

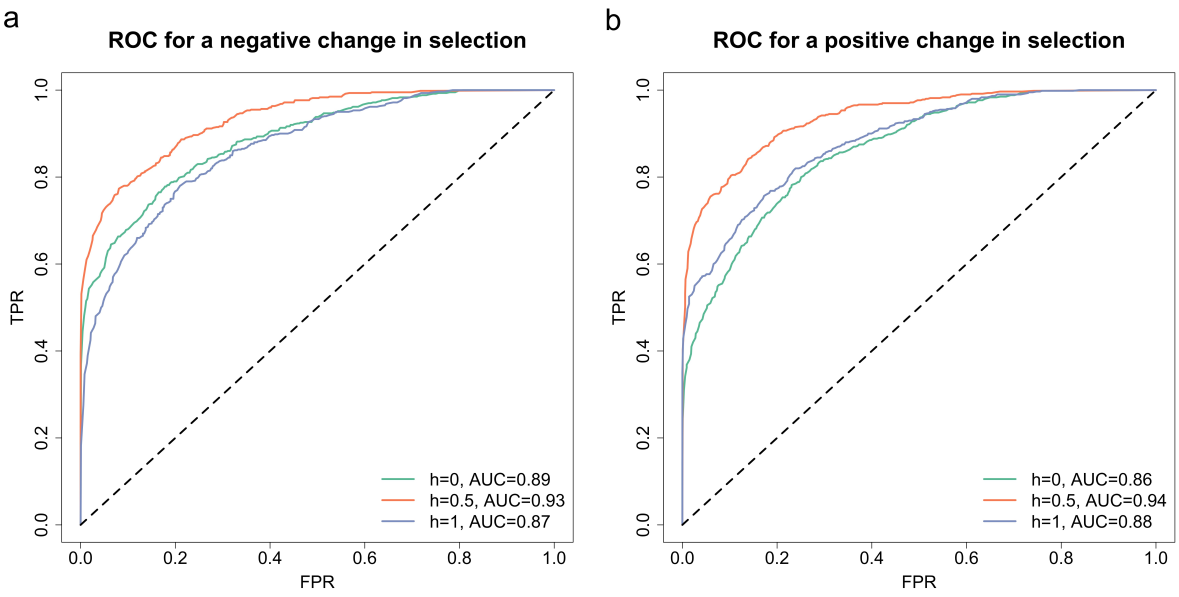

Figure S4: ROC curves for testing a change in selection across different gene actions and selection scenarios with the parameters $\phi = 0.85$ and $\psi = 1$ (*i.e.*, scenario D in Table 1). For each combination, we repeatedly run our procedure on 200 simulated datasets. The AUC value for each curve is summarised. Selection scenarios (scenarios 1–9) are described in Table 2. ROC curves for (a) a negative change in selection and (b) a positive change in selection.

Dom. param. h	Sel. coeff. s^- Bias	Sel. coeff. s^- RMSE	Sel. coeff. s^+ Bias	Sel. coeff. s^+ RMSE
0.0	−0.00254	0.01612	−0.01057	0.04167
0.5	0.00022	0.00348	0.00073	0.00595
1.0	0.00614	0.01508	0.00219	0.01537

(a) Scenario 1: $s^- < 0$ and $s^+ < s^-$.

Dom. param. h	Sel. coeff. s^- Bias	Sel. coeff. s^- RMSE	Sel. coeff. s^+ Bias	Sel. coeff. s^+ RMSE
0.0	−0.00231	0.01663	−0.00909	0.03651
0.5	0.00067	0.00369	−0.00018	0.00592
1.0	0.00137	0.00633	0.00014	0.00678

(b) Scenario 2: $s^- < 0$ and $s^+ = s^-$.

Dom. param. h	Sel. coeff. s^- Bias	Sel. coeff. s^- RMSE	Sel. coeff. s^+ Bias	Sel. coeff. s^+ RMSE
0.0	−0.00326	0.01638	−0.00951	0.03096
0.5	0.00128	0.00499	−0.00165	0.00593
1.0	0.00128	0.00745	0.00026	0.01085

(c) Scenario 3: $s^- < 0$ and $s^+ > s^-$.

Dom. param. h	Sel. coeff. s^- Bias	Sel. coeff. s^- RMSE	Sel. coeff. s^+ Bias	Sel. coeff. s^+ RMSE
0.0	−0.00181	0.00809	−0.00147	0.01473
0.5	0.00064	0.00362	−0.00027	0.00508
1.0	0.00334	0.00986	0.00179	0.00866

(d) Scenario 4: $s^- = 0$ and $s^+ < s^-$.

Dom. param. h	Sel. coeff. s^- Bias	Sel. coeff. s^- RMSE	Sel. coeff. s^+ Bias	Sel. coeff. s^+ RMSE
0.0	−0.00206	0.01300	−0.00080	0.00798
0.5	0.00015	0.00422	0.00040	0.00330
1.0	0.00137	0.00727	0.00173	0.01050

(e) Scenario 5: $s^- = 0$ and $s^+ = s^-$.

Table S6: Mean bias and RMSE in MAP estimates of the selection coefficients across different gene actions and selection scenarios with the parameters $\phi = 0.85$ and $\psi = 1$ (*i.e.*, scenario D in Table 1), corresponding to Figure S3. Mean bias and RMSE are calculated with 200 replicates for each combination. Selection scenarios (scenarios 1–9) are described in Table 2.

Dom. param. h	Sel. coeff. s^- Bias	Sel. coeff. s^- RMSE	Sel. coeff. s^+ Bias	Sel. coeff. s^+ RMSE
0.0	−0.00268	0.00944	−0.00176	0.00845
0.5	−0.00061	0.00411	0.00021	0.00548
1.0	0.00089	0.00718	0.00222	0.01564

(f) Scenario 6: $s^- = 0$ and $s^+ > s^-$.

Dom. param. h	Sel. coeff. s^- Bias	Sel. coeff. s^- RMSE	Sel. coeff. s^+ Bias	Sel. coeff. s^+ RMSE
0.0	−0.00139	0.00518	−0.00044	0.01025
0.5	−0.00128	0.00529	0.00021	0.00593
1.0	0.00120	0.01551	0.01203	0.03144

(g) Scenario 7: $s^- > 0$ and $s^+ < s^-$.

Dom. param. h	Sel. coeff. s^- Bias	Sel. coeff. s^- RMSE	Sel. coeff. s^+ Bias	Sel. coeff. s^+ RMSE
0.0	−0.00104	0.00632	−0.00044	0.00891
0.5	−0.00066	0.00389	0.00001	0.00547
1.0	0.00188	0.01367	0.00719	0.03706

(h) Scenario 8: $s^- > 0$ and $s^+ = s^-$.

Dom. param. h	Sel. coeff. s^- Bias	Sel. coeff. s^- RMSE	Sel. coeff. s^+ Bias	Sel. coeff. s^+ RMSE
0.0	−0.00471	0.01205	−0.00043	0.00819
0.5	−0.00019	0.00350	−0.00006	0.00687
1.0	0.00135	0.01567	0.00558	0.03726

(i) Scenario 9: $s^- > 0$ and $s^+ > s^-$.

Table S6: Mean bias and RMSE in MAP estimates of the selection coefficients across different gene actions and selection scenarios with the parameters $\phi = 0.85$ and $\psi = 1$ (*i.e.*, scenario D in Table 1), corresponding to Figure S3, continued.

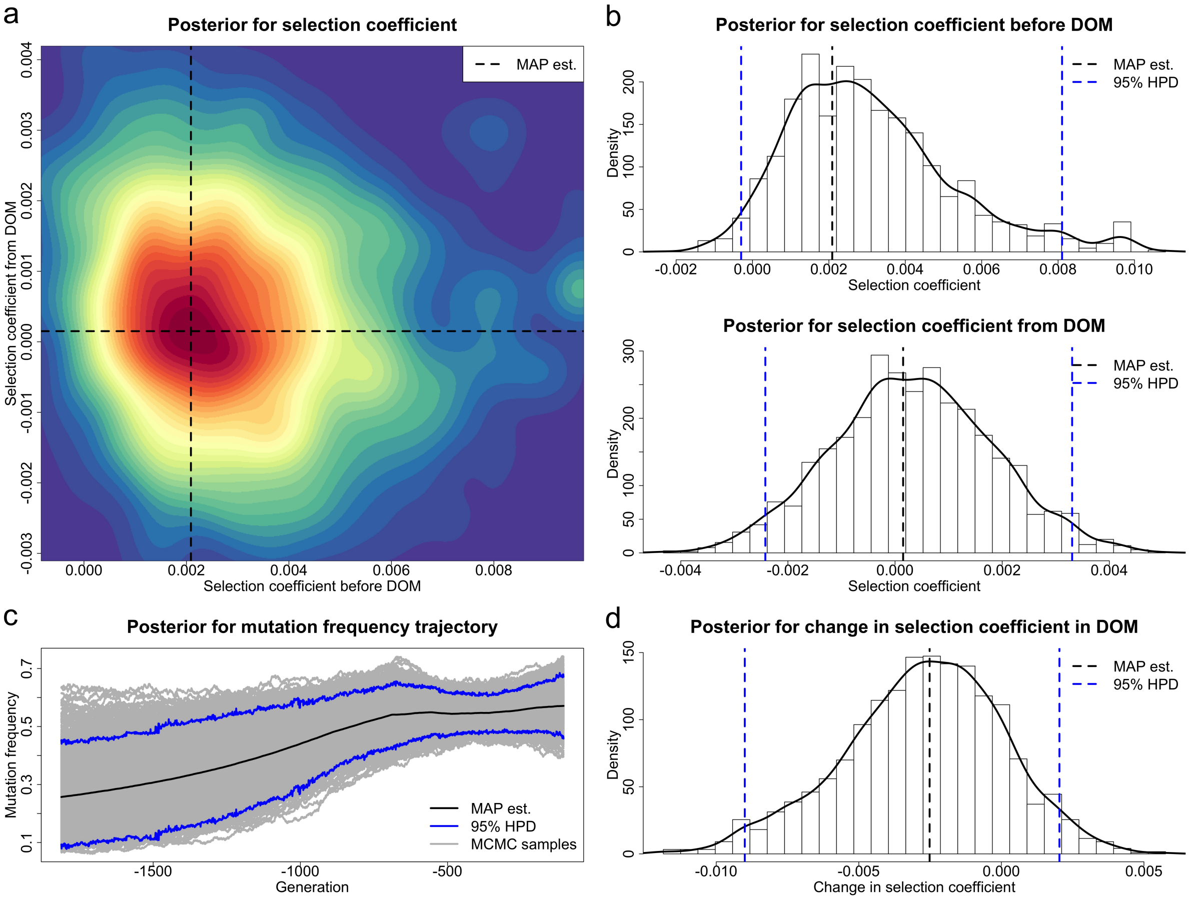

Figure S5: Posteriors for the selection coefficients of the *ASIP* mutation before and from horse domestication (starting from 3500 BC) and the underlying frequency trajectory of the *ASIP* mutation in the population. The demographic history is fixed at $N = 16000$, and the samples drawn before 12500 BC are excluded. DOM stands for domestication. (a) Joint posterior for the selection coefficients s^- and s^+. (b) Marginal posteriors for the selection coefficients s^- and s^+. (c) Posterior for the underlying frequency trajectory of the *ASIP* mutation. (d) Posterior for the selection change Δs.

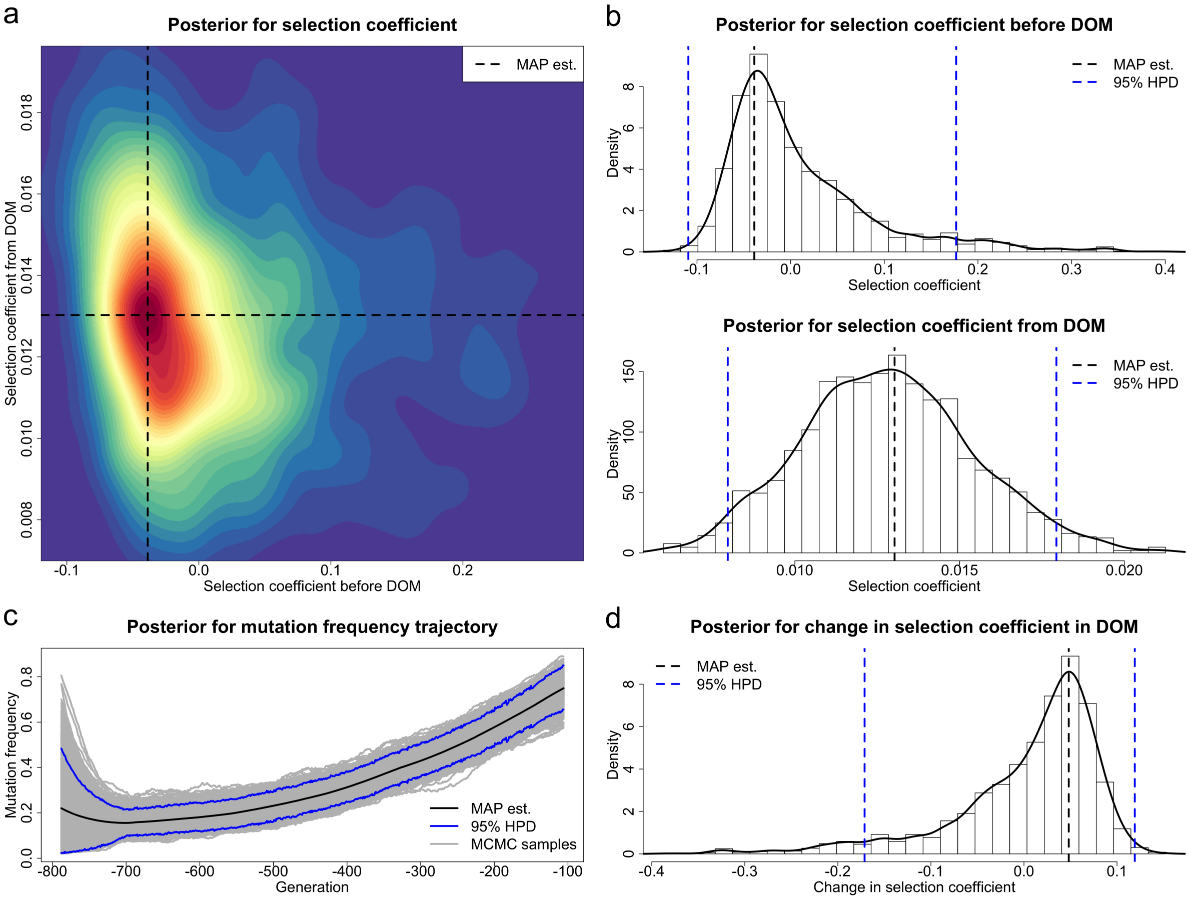

Figure S6: Posteriors for the selection coefficients of the *MC1R* mutation before and from horse domestication (starting from 3500 BC) and the underlying frequency trajectory of the *MC1R* mutation in the population. The demographic history is fixed at $N = 16000$, and the samples drawn before 4300 BC are excluded. DOM stands for domestication. (a) Joint posterior for the selection coefficients s^- and s^+. (b) Marginal posteriors for the selection coefficients s^- and s^+. (c) Posterior for the underlying frequency trajectory of the *MC1R* mutation. (d) Posterior for the selection change Δs.

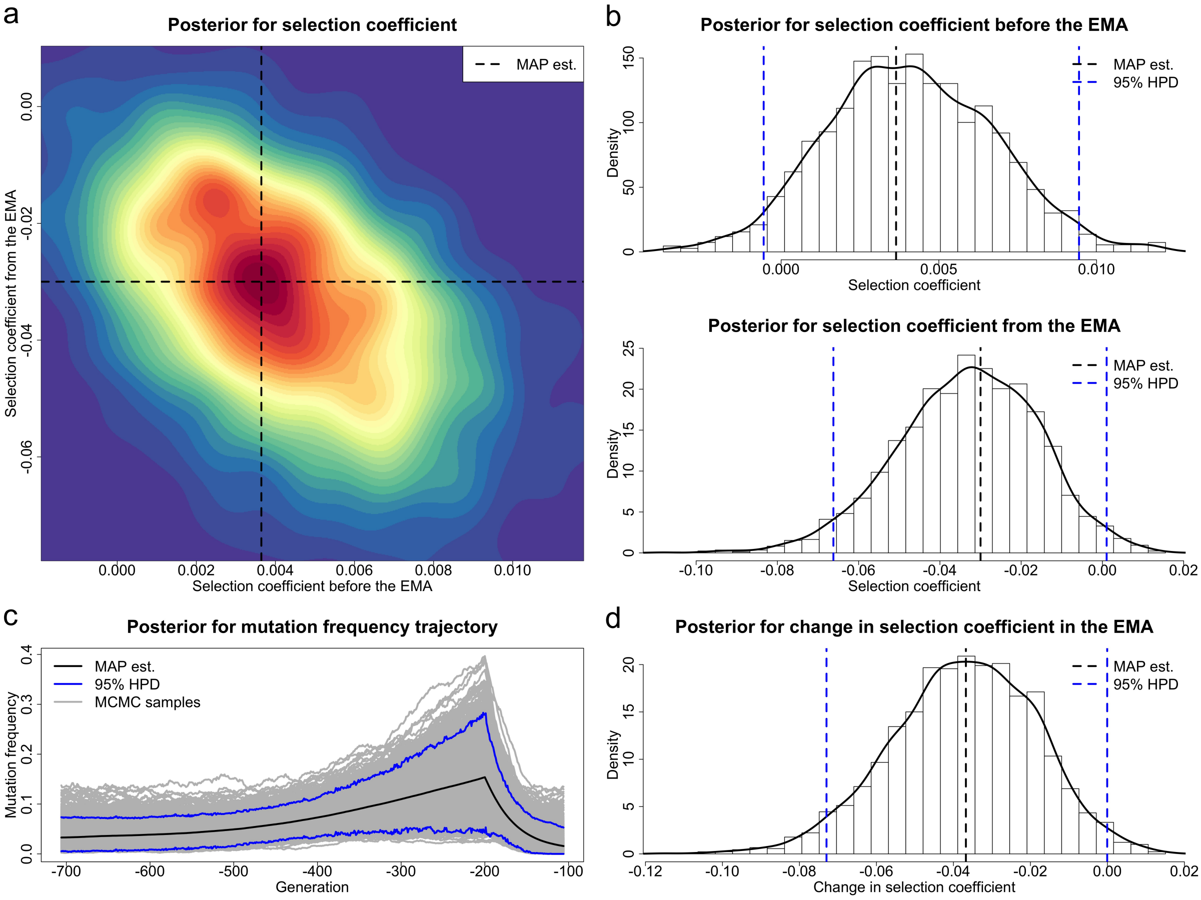

Figure S7: Posteriors for the selection coefficients of the *KIT13* mutation before and from the Middle Ages (starting from AD 400) and the underlying frequency trajectory of the *KIT13* mutation in the population. The demographic history is fixed at $N = 16000$, and the samples drawn before 3645 BC are excluded. EMA stands for Early Middle Ages. (a) Joint posterior for the selection coefficients s^- and s^+. (b) Marginal posteriors for the selection coefficients s^- and s^+. (c) Posterior for the underlying frequency trajectory of the *KIT13* mutation. (d) Posterior for the selection change Δs.

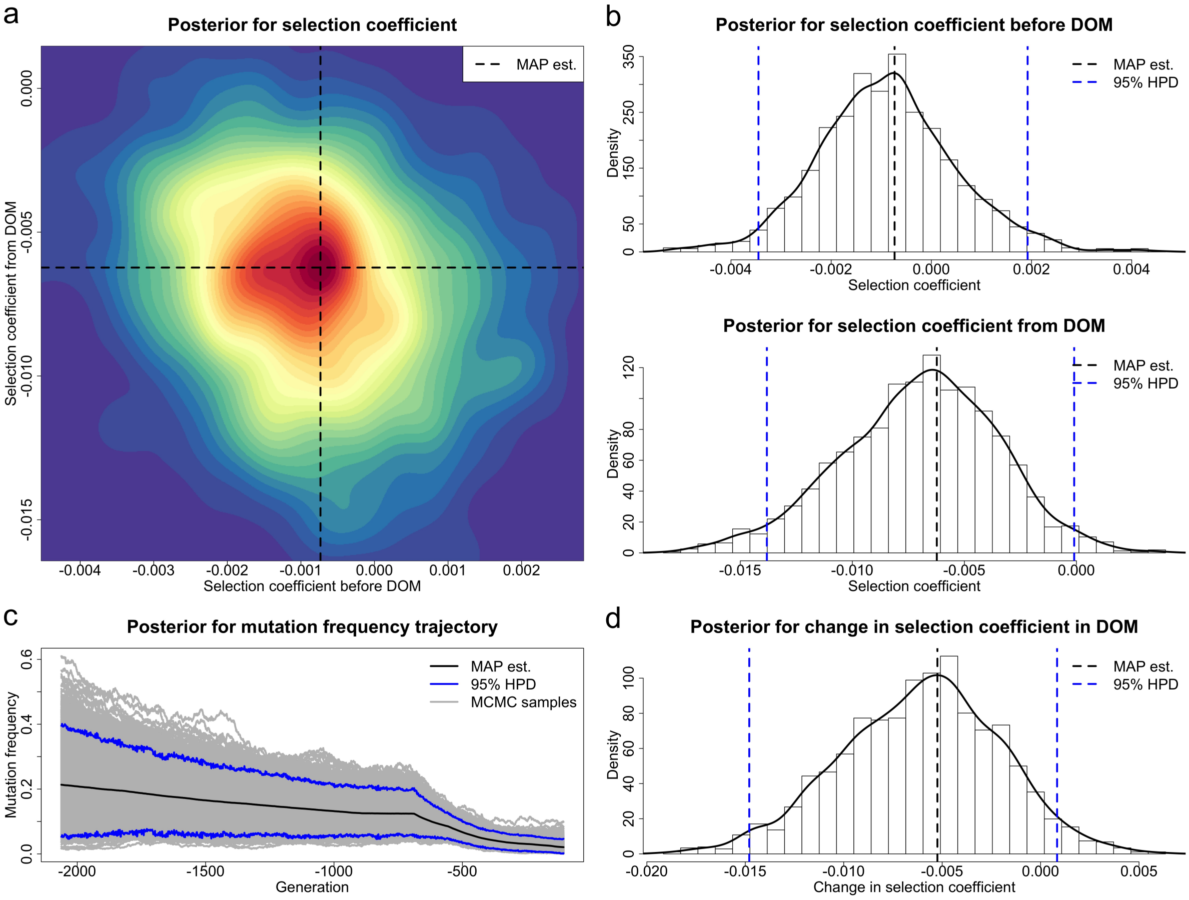

Figure S8: Posteriors for the selection coefficients of the *TRPM1* mutation before and from horse domestication (starting from 3500 BC) and the underlying frequency trajectory of the *TRPM1* mutation in the population. The demographic history is fixed at $N = 16000$, and the samples drawn before 14500 BC are excluded. DOM stands for domestication. (a) Joint posterior for the selection coefficients s^- and s^+. (b) Marginal posteriors for the selection coefficients s^- and s^+. (c) Posterior for the underlying frequency trajectory of the *TRPM1* mutation. (d) Posterior for the selection change Δs.

Gene	Event	Sel. coeff.	MAP est.	95% HPD	Prob. for -ve.	Prob. for +ve.
		s^-	0.00209	[−0.00030, 0.00810]	0.0370	0.9630
ASIP	DOM	s^+	0.00015	[−0.00242, 0.00331]	0.4195	0.5805
		Δs	−0.00252	[−0.00900, 0.00204]	0.8455	0.1545
		s^-	−0.03878	[−0.10923, 0.17673]	0.5920	0.4080
MC1R	DOM	s^+	0.01303	[0.00796, 0.01794]	0.0000	1.0000
		Δs	0.04813	[−0.17128, 0.11910]	0.3510	0.6490
		s^-	0.00364	[−0.00055, 0.00944]	0.0525	0.9475
KIT13	EMA	s^+	−0.03000	[−0.06621, 0.00104]	0.9805	0.0195
		Δs	−0.03676	[−0.07297, −0.00005]	0.9840	0.0160
		s^-	−0.00073	[−0.00345, 0.00192]	0.7635	0.2365
TRPM1	DOM	s^+	−0.00623	[−0.01381, −0.00010]	0.9830	0.0170
		Δs	−0.00524	[−0.01480, 0.00084]	0.9440	0.0560

Table S7: MAP estimates of the selection coefficients and their changes with their 95% HPD intervals, as well as posterior probabilities for negative selection/change and positive selection/change, for *ASIP*, *MC1R*, *KIT13* and *TRPM1*, corresponding to Figures S5–S8. Prob. for -ve. and +ve. stand for posterior probabilities for negative selection/change and positive selection/change, respectively. The demographic history is fixed at $N = 16000$. DOM and EMA stand for domestication and Early Middle Ages, respectively.

Method	Data	Gene	s	h	N	Reject $s = 0$	Note
Bollback et al. (2008)	Ludwig et al. (2009)	*ASIP*	$s > 0$	codominance	constant	yes	5 years/generation, 6 sampling times
		MC1R	$s > 0$	codominance	constant	yes	5 years/generation, 6 sampling times
Malaspinas et al. (2012)	Ludwig et al. (2009)	*ASIP*	$s < 0$	recessive	constant	no	5 years/generation, 6 sampling times
Steinrücken et al. (2014)	Ludwig et al. (2009)	*ASIP*	$s > 0$	overdominance	constant	yes	5 years/generation, 6 sampling times
		MC1R	$s > 0$	overdominance	constant	yes	5 years/generation, 6 sampling times
Schraiber et al. (2016)	Ludwig et al. (2009)	*ASIP*	$s > 0$	codominance	time-varying	yes	8 years/generation, 6 sampling times
		MC1R	$s > 0$	overdominance	time-varying	yes	8 years/generation, 6 sampling times
He et al. (2020a)	Wutke et al. (2018)	*KIT13*	$s > 0$	dominant	constant	no	8 years/generation, 9 sampling times
He et al. (2020b)	Wutke et al. (2018)	*ASIP*	$s > 0$	recessive	time-varying	yes	8 years/generation, 65 sampling times
		MC1R	$s > 0$	recessive	time-varying	yes	8 years/generation, 65 sampling times

Table S8: Summary of the results for selection acting on the horse coat colouration loci produced from ancient horse samples in earlier studies. Among these studies, except for Steinrücken et al. (2014) and Schraiber et al. (2016), the dominance parameter h was prespecified, and except for Malaspinas et al. (2012), the population size N was prespecified. The different constant population size was adopted across different studies, and the same time-varying population size reported in Der Sarkissian et al. (2015) was used in Schraiber et al. (2016) and He et al. (2020b). The selection coefficient of *KIT13* in He et al. (2020a) was co-estimated with that of *KIT16* in consideration of genetic recombination and local linkage. For comparative purposes, the estimates for the selection coefficients are all converted into the same selection model parameterisation adopted in our work. The selection coefficient s represents the strength of selection acting on the homozygous double mutant genotype except for $ASIP$ in Steinrücken et al. (2014), where s denotes the strength of selection acting on the heterozygous genotype since their estimate for the homozygous double mutant genotype is 0. Note that the dataset of Wutke et al. (2018) consists of the ancient horse samples from Ludwig et al. (2009), Pruvost et al. (2011) and Wutke et al. (2018).

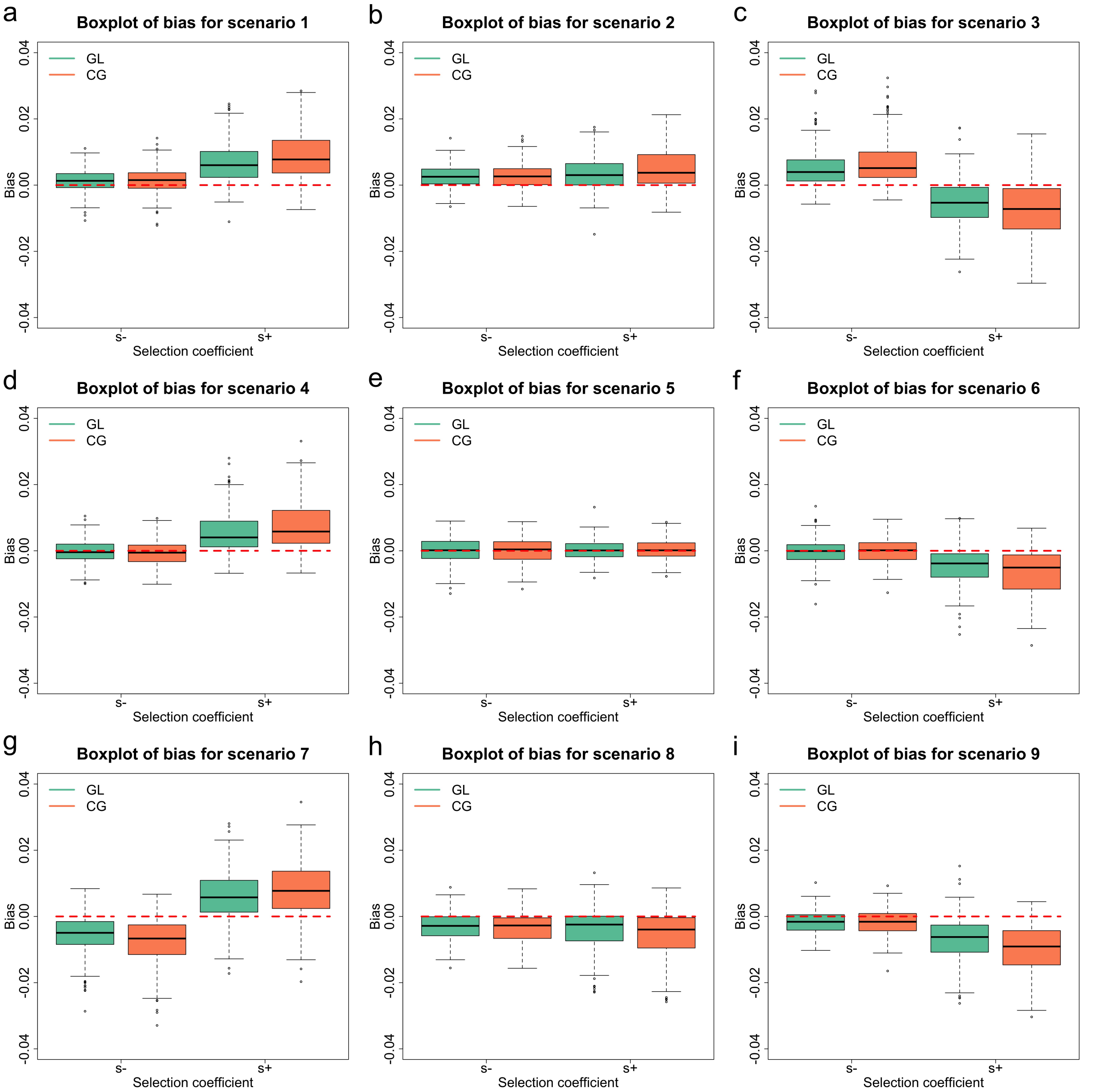

(a) Scenario A: $\phi = 0.75$ and $\psi = 0.5$.

Figure S9: Empirical distributions for the bias in MAP estimates of the selection coefficients across different input types, data qualities and selection scenarios. For each combination, we repeatedly run our procedure on 200 simulated datasets. Data qualities (scenarios A–F) are described in Table 1, and selection scenarios (scenarios 1–9) are described in Table 2. GL and CG are shorthands separately for genotype likelihood and called genotype.

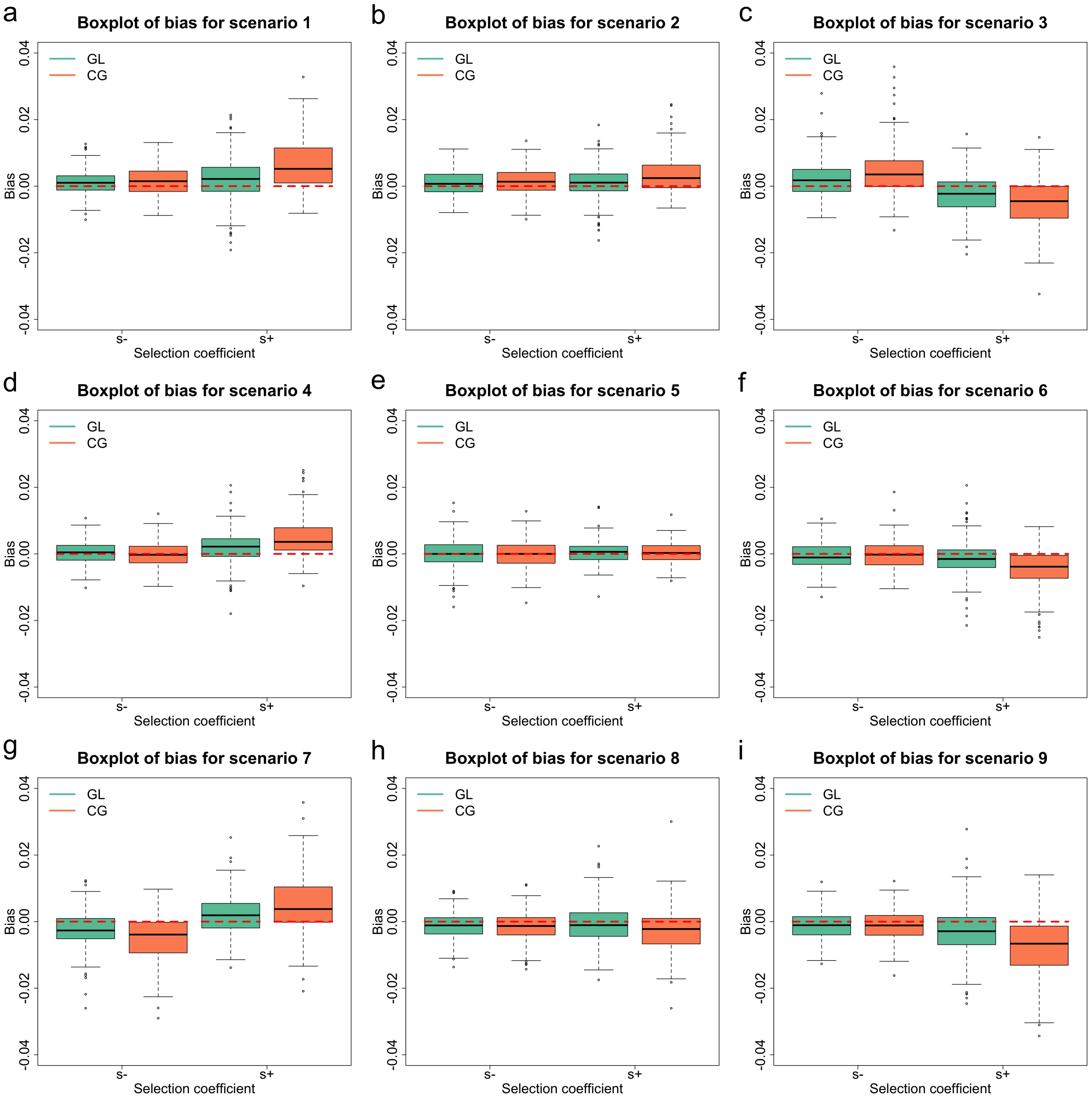

(b) Scenario B: $\phi = 0.75$ and $\psi = 1$.

Figure S9: Empirical distributions for the bias in MAP estimates of the selection coefficients across different input types, data qualities and selection scenarios, continued.

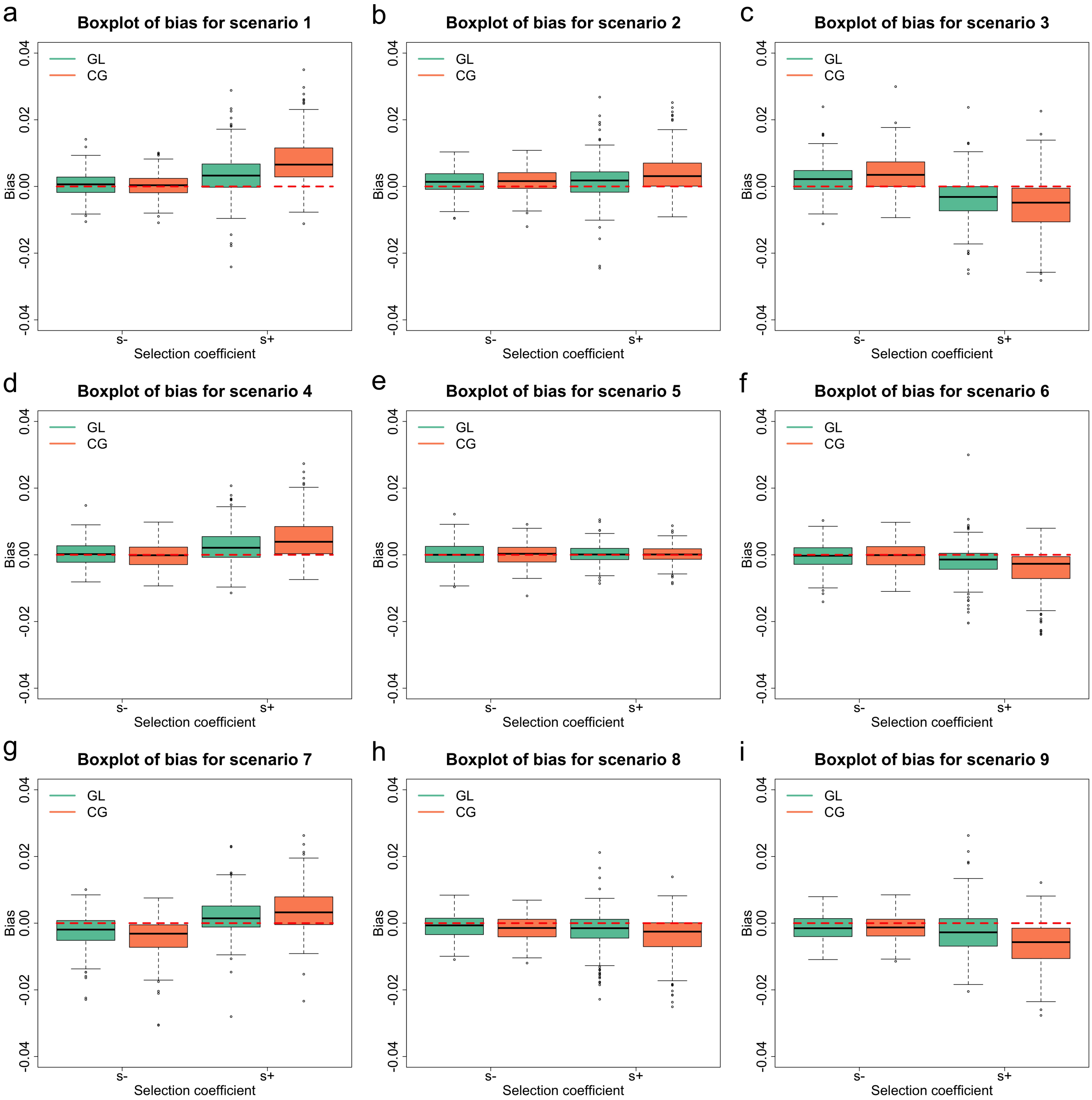

(c) Scenario C: $\phi = 0.85$ and $\psi = 0.5$.

Figure S9: Empirical distributions for the bias in MAP estimates of the selection coefficients across different input types, data qualities and selection scenarios, continued.

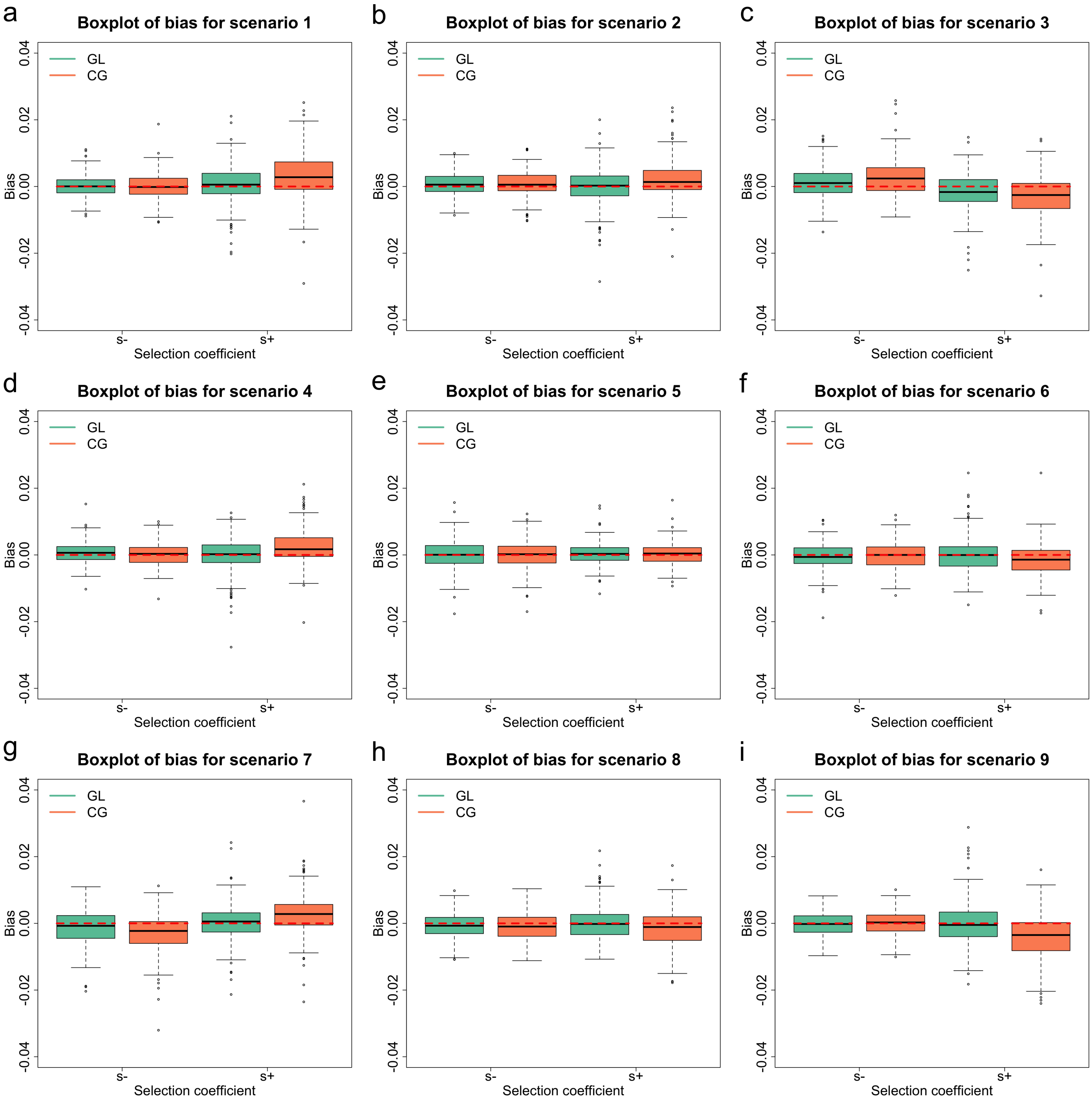

(d) Scenario D: $\phi = 0.85$ and $\psi = 1$.

Figure S9: Empirical distributions for the bias in MAP estimates of the selection coefficients across different input types, data qualities and selection scenarios, continued.

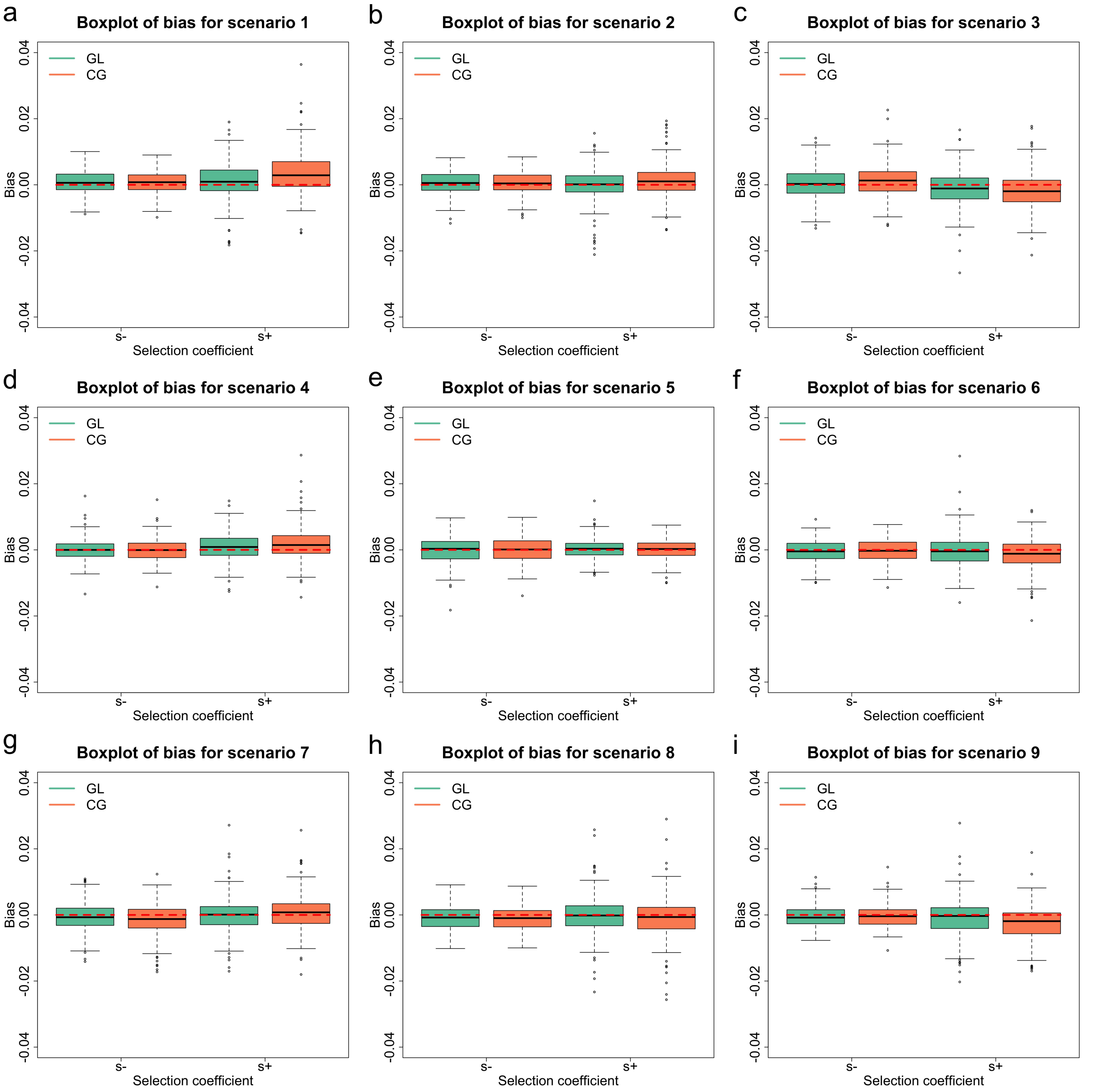

(e) Scenario E: $\phi = 0.95$ and $\psi = 0.5$.

Figure S9: Empirical distributions for the bias in MAP estimates of the selection coefficients across different input types, data qualities and selection scenarios, continued.

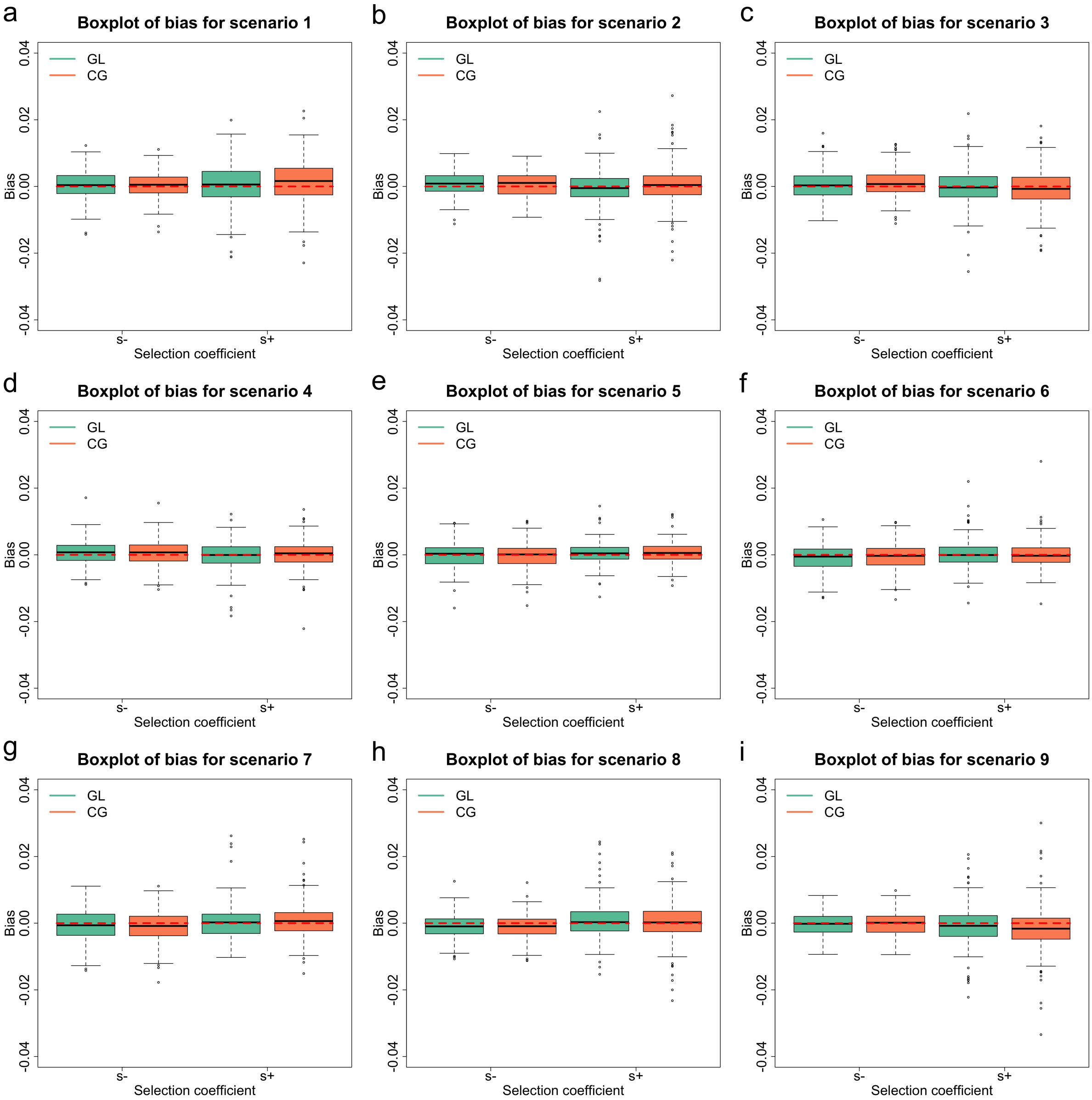

(f) Scenario F: $\phi = 0.95$ and $\psi = 1$.

Figure S9: Empirical distributions for the bias in MAP estimates of the selection coefficients across different input types, data qualities and selection scenarios, continued.

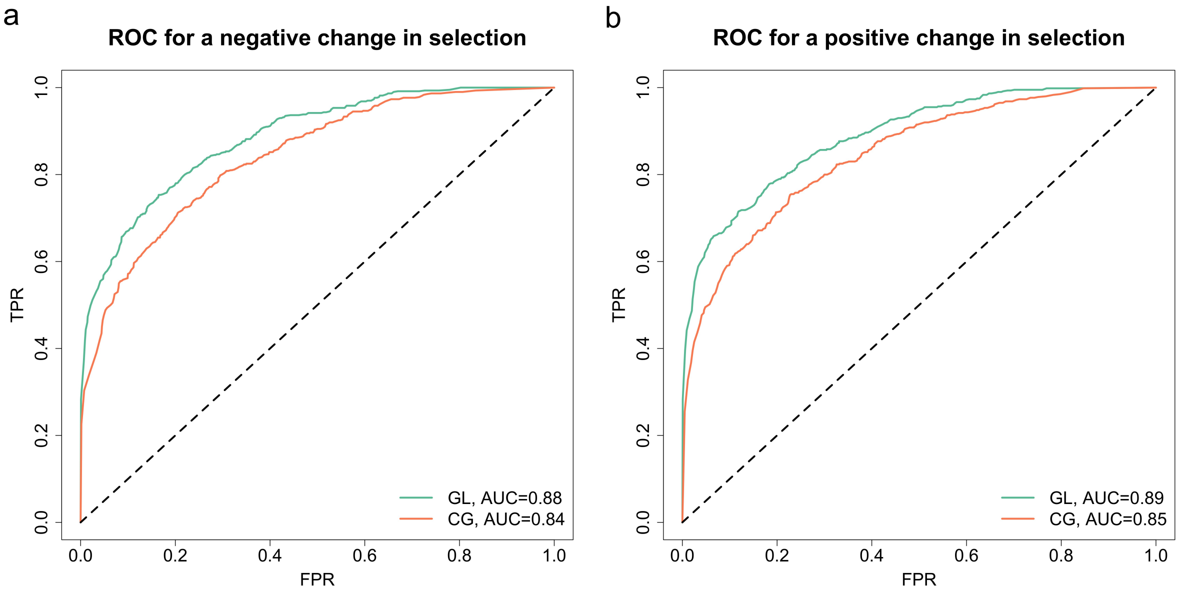

(a) Scenario A: $\phi = 0.75$ and $\psi = 0.5$.

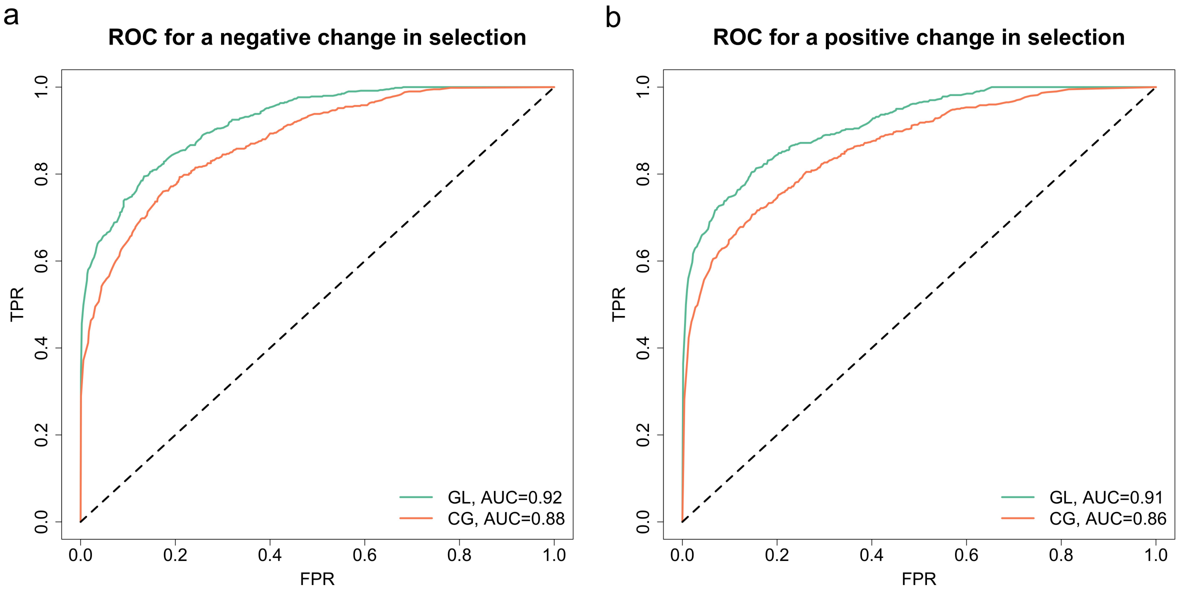

(b) Scenario B: $\phi = 0.75$ and $\psi = 1$.

Figure S10: ROC curves for testing a change in selection across different input types, data qualities and selection scenarios. For each combination, we repeatedly run our procedure on 200 simulated datasets. The AUC value for each curve is summarised. Data qualities (scenarios A–F) are described in Table 1, and selection scenarios (scenarios 1–9) are described in Table 2. GL and CG are shorthands separately for genotype likelihood and called genotype.

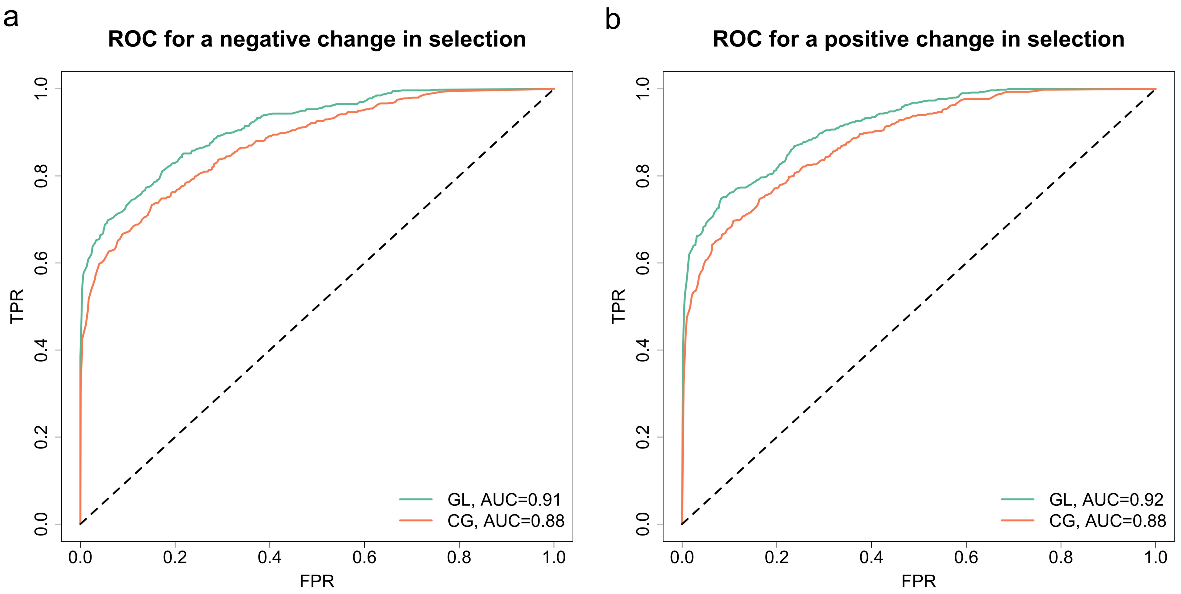

(c) Scenario C: $\phi = 0.85$ and $\psi = 0.5$.

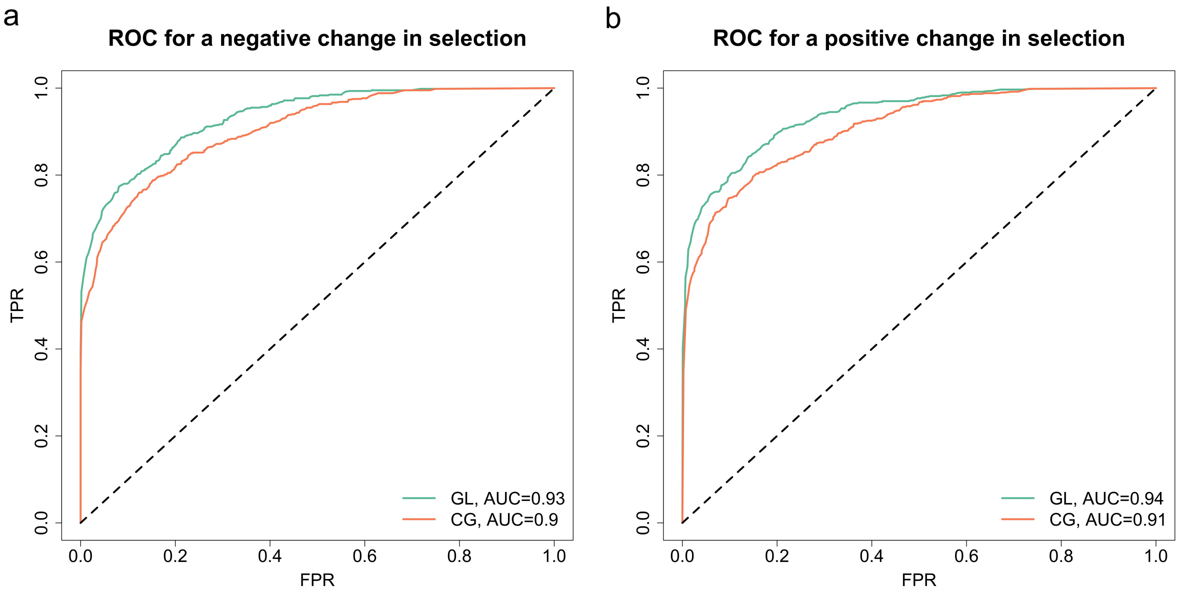

(d) Scenario D: $\phi = 0.85$ and $\psi = 1$.

Figure S10: ROC curves for testing a change in selection across different input types, data qualities and selection scenarios, continued.

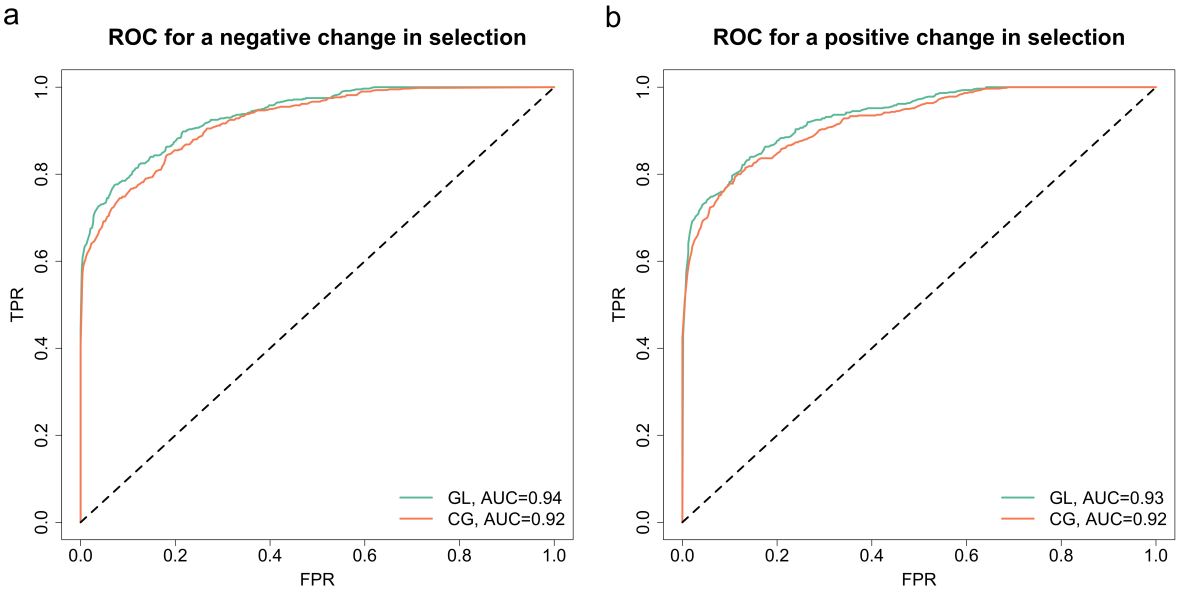

(e) Scenario E: $\phi = 0.95$ and $\psi = 0.5$.

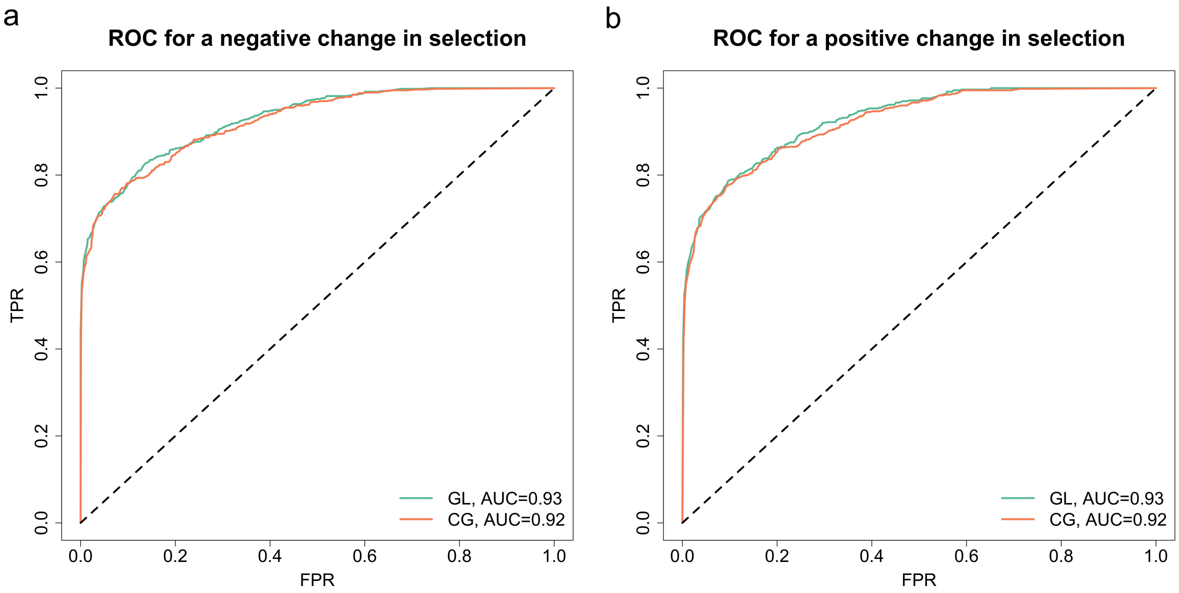

(f) Scenario F: $\phi = 0.95$ and $\psi = 1$.

Figure S10: ROC curves for testing a change in selection across different input types, data qualities and selection scenarios, continued.

Input	Scenario	Sel. coeff. s^-		Sel. coeff. s^+	
		Bias	RMSE	Bias	RMSE
GL	A	0.00126	0.00391	0.00684	0.00952
	B	0.00124	0.00464	0.00229	0.00708
	C	0.00054	0.00399	0.00378	0.00806
	D	0.00022	0.00348	0.00073	0.00595
	E	0.00081	0.00354	0.00104	0.00603
	F	0.00043	0.00402	0.00053	0.00618
CG	A	0.00142	0.00427	0.00920	0.01185
	B	0.00157	0.00467	0.00671	0.01028
	C	0.00037	0.00380	0.00779	0.01082
	D	0.00008	0.00384	0.00357	0.00754
	E	0.00061	0.00358	0.00364	0.00753
	F	0.00038	0.00374	0.00153	0.00659

(a) Scenario 1: $s^- < 0$ and $s^+ < s^-$.

Input	Scenario	Sel. coeff. s^-		Sel. coeff. s^+	
		Bias	RMSE	Bias	RMSE
GL	A	0.00258	0.00438	0.00358	0.00646
	B	0.00111	0.00411	0.00100	0.00507
	C	0.00131	0.00387	0.00167	0.00627
	D	0.00067	0.00369	−0.00018	0.00592
	E	0.00042	0.00372	−0.00005	0.00541
	F	0.00056	0.00358	−0.00051	0.00577
CG	A	0.00279	0.00467	0.00502	0.00790
	B	0.00159	0.00439	0.00333	0.00646
	C	0.00148	0.00403	0.00412	0.00733
	D	0.00080	0.00399	0.00224	0.00624
	E	0.00051	0.00368	0.00144	0.00548
	F	0.00053	0.00359	0.00058	0.00594

(b) Scenario 2: $s^- < 0$ and $s^+ = s^-$.

Table S9: Mean bias and RMSE in MAP estimates of the selection coefficients across different input types, data qualities and selection scenarios, corresponding to Figure S9. Mean bias and RMSE are calculated with 200 replicates for each combination. Data qualities (scenarios A–F) are described in Table 1, and selection scenarios (scenarios 1–9) are described in Table 2. GL and CG are shorthands separately for genotype likelihood and called genotype.

Input	Scenario	Sel. coeff. s^- Bias	Sel. coeff. s^- RMSE	Sel. coeff. s^+ Bias	Sel. coeff. s^+ RMSE
GL	A	0.00503	0.00777	−0.00537	0.00896
	B	0.00214	0.00585	−0.00263	0.00638
	C	0.00244	0.00547	−0.00331	0.00725
	D	0.00128	0.00499	−0.00165	0.00593
	E	0.00038	0.00462	−0.00104	0.00558
	F	0.00052	0.00432	−0.00018	0.00563
CG	A	0.00690	0.00981	−0.00737	0.01115
	B	0.00455	0.00846	−0.00533	0.00914
	C	0.00409	0.00702	−0.00525	0.00928
	D	0.00270	0.00614	−0.00291	0.00695
	E	0.00130	0.00503	−0.00173	0.00586
	F	0.00106	0.00432	−0.00169	0.01681

(c) Scenario 3: $s^- < 0$ and $s^+ > s^-$.

Input	Scenario	Sel. coeff. s^- Bias	Sel. coeff. s^- RMSE	Sel. coeff. s^+ Bias	Sel. coeff. s^+ RMSE
GL	A	−0.00023	0.00347	0.00551	0.00834
	B	0.00028	0.00348	0.00191	0.00528
	C	0.00022	0.00366	0.00265	0.00571
	D	0.00064	0.00362	−0.00027	0.00508
	E	−0.00001	0.00338	0.00079	0.00432
	F	0.00063	0.00368	−0.00035	0.00424
CG	A	−0.00058	0.00366	0.00772	0.01087
	B	−0.00013	0.00381	0.00483	0.00753
	C	−0.00012	0.00360	0.00496	0.00793
	D	0.00023	0.00374	0.00243	0.00598
	E	−0.00006	0.00336	0.00183	0.00546
	F	0.00057	0.00371	0.00022	0.00412

(d) Scenario 4: $s^- = 0$ and $s^+ < s^-$.

Table S9: Mean bias and RMSE in MAP estimates of the selection coefficients across different input types, data qualities and selection scenarios, corresponding to Figure S9, continued.

Input	Scenario	Sel. coeff. s^- Bias	Sel. coeff. s^- RMSE	Sel. coeff. s^+ Bias	Sel. coeff. s^+ RMSE
GL	A	0.00020	0.00389	0.00026	0.00300
	B	−0.00001	0.00437	0.00044	0.00337
	C	0.00015	0.00368	0.00022	0.00292
	D	0.00015	0.00422	0.00040	0.00330
	E	−0.00003	0.00391	0.00028	0.00301
	F	−0.00007	0.00380	0.00040	0.00328
CG	A	0.00014	0.00370	0.00023	0.00285
	B	−0.00011	0.00425	0.00036	0.00315
	C	0.00016	0.00348	0.00017	0.00277
	D	0.00017	0.00421	0.00033	0.00326
	E	0.00002	0.00380	0.00025	0.00293
	F	−0.00022	0.00375	0.00062	0.00314

(e) Scenario 5: $s^- = 0$ and $s^+ = s^-$.

Input	Scenario	Sel. coeff. s^- Bias	Sel. coeff. s^- RMSE	Sel. coeff. s^+ Bias	Sel. coeff. s^+ RMSE
GL	A	−0.00031	0.00368	−0.00460	0.00722
	B	−0.00084	0.00409	−0.00134	0.00548
	C	−0.00054	0.00394	−0.00195	0.00571
	D	−0.00061	0.00411	0.00021	0.00548
	E	−0.00060	0.00365	−0.00034	0.00494
	F	−0.00086	0.00397	0.00037	0.00427
CG	A	0.00000	0.00354	−0.00656	0.00952
	B	−0.00033	0.00420	−0.00453	0.00756
	C	−0.00032	0.00385	−0.00422	0.00749
	D	−0.00029	0.00408	−0.00160	0.00523
	E	−0.00039	0.00371	−0.00142	0.00497
	F	−0.00071	0.00390	−0.00001	0.00422

(f) Scenario 6: $s^- = 0$ and $s^+ > s^-$.

Table S9: Mean bias and RMSE in MAP estimates of the selection coefficients across different input types, data qualities and selection scenarios, corresponding to Figure S9, continued.

Input	Scenario	Sel. coeff. s^- Bias	Sel. coeff. s^- RMSE	Sel. coeff. s^+ Bias	Sel. coeff. s^+ RMSE
GL	A	−0.00558	0.00834	0.00585	0.00951
	B	−0.00259	0.00619	0.00200	0.00611
	C	−0.00265	0.00580	0.00195	0.00624
	D	−0.00128	0.00529	0.00021	0.00593
	E	−0.00064	0.00452	0.00002	0.00569
	F	−0.00067	0.00475	0.00034	0.00552
CG	A	−0.00774	0.01066	0.00825	0.01236
	B	−0.00506	0.00837	0.00507	0.00953
	C	−0.00432	0.00741	0.00406	0.00805
	D	−0.00304	0.00670	0.00272	0.00693
	E	−0.00158	0.00522	0.00083	0.00564
	F	−0.00111	0.00480	0.00078	0.00561

(g) Scenario 7: $s^- > 0$ and $s^+ < s^-$.

Input	Scenario	Sel. coeff. s^- Bias	Sel. coeff. s^- RMSE	Sel. coeff. s^+ Bias	Sel. coeff. s^+ RMSE
GL	A	−0.00295	0.00521	−0.00383	0.00721
	B	−0.00131	0.00415	−0.00093	0.00596
	C	−0.00104	0.00371	−0.00219	0.00628
	D	−0.00066	0.00389	0.00001	0.00547
	E	−0.00090	0.00385	−0.00033	0.00598
	F	−0.00092	0.00359	0.00079	0.00572
CG	A	−0.00342	0.00562	−0.00537	0.00862
	B	−0.00164	0.00476	−0.00325	0.00731
	C	−0.00161	0.00409	−0.00407	0.00740
	D	−0.00093	0.00397	−0.00170	0.00602
	E	−0.00100	0.00380	−0.00113	0.00633
	F	−0.00105	0.00372	0.00014	0.00602

(h) Scenario 8: $s^- > 0$ and $s^+ = s^-$.

Table S9: Mean bias and RMSE in MAP estimates of the selection coefficients across different input types, data qualities and selection scenarios, corresponding to Figure S9, continued.

Input	Scenario	Sel. coeff. s^- Bias	Sel. coeff. s^- RMSE	Sel. coeff. s^+ Bias	Sel. coeff. s^+ RMSE
GL	A	-0.00167	0.00412	-0.00713	0.00982
	B	-0.00124	0.00434	-0.00294	0.00792
	C	-0.00142	0.00380	-0.00284	0.00744
	D	-0.00019	0.00350	-0.00006	0.00687
	E	-0.00047	0.00332	-0.00082	0.00597
	F	-0.00038	0.00365	-0.00068	0.00612
CG	A	-0.00183	0.00450	-0.00984	0.01226
	B	-0.00128	0.00454	-0.00817	0.01194
	C	-0.00136	0.00389	-0.00671	0.00972
	D	0.00017	0.00358	-0.00397	0.00885
	E	-0.00034	0.00349	-0.00268	0.00615
	F	-0.00034	0.00360	-0.00171	0.00719

(i) Scenario 9: $s^- > 0$ and $s^+ > s^-$.

Table S9: Mean bias and RMSE in MAP estimates of the selection coefficients across different input types, data qualities and selection scenarios, corresponding to Figure S9, continued.

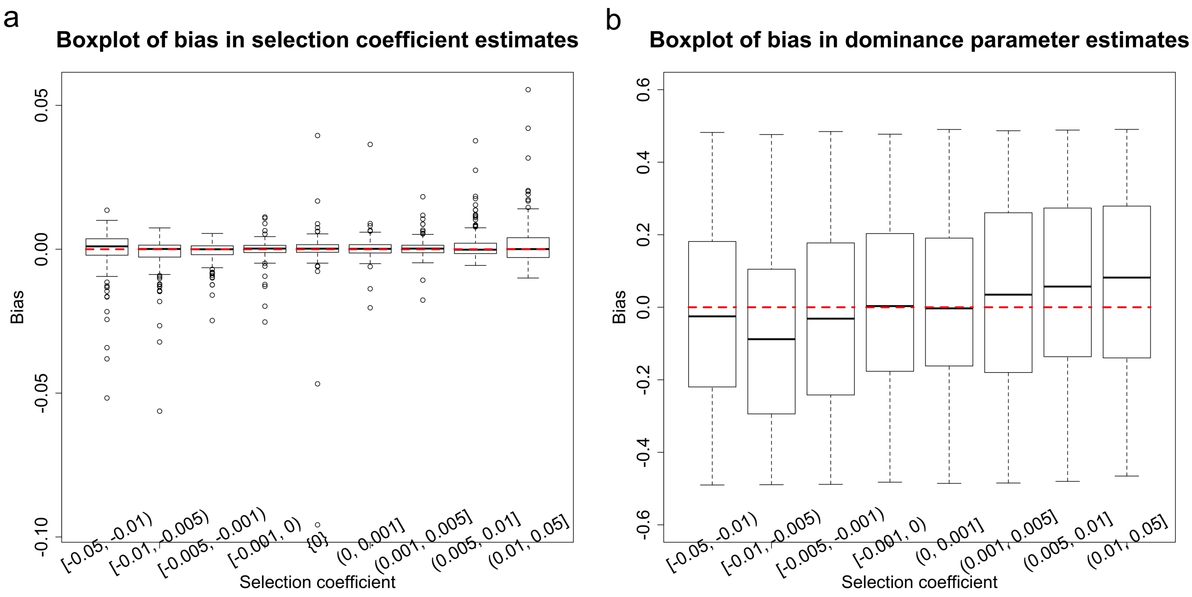

Figure S11: Empirical distributions for the bias in MAP estimates of the selection coefficient and dominance parameter across different ranges of the selection coefficient s with the parameters $\phi = 0.85$ and $\psi = 1$ (*i.e.*, scenario D in Table 1. The simulated datasets for each range of the selection coefficient are the same as those in Figure 4. Boxplots for the bias in estimation of (a) the selection coefficient and (b) the dominance parameter.

Sel. coeff. s	Sel. coeff. s		Dom. param. h	
	Bias	RMSE	Bias	RMSE
$s \in [-0.050, -0.010)$	-0.00035	0.00734	-0.01851	0.25650
$s \in [-0.010, -0.005)$	-0.00163	0.00658	-0.08433	0.26988
$s \in [-0.005, -0.001)$	-0.00087	0.00367	-0.03049	0.27109
$s \in [-0.001, 0)$	-0.00016	0.00351	0.00004	0.25984
$s \in \{0\}$	-0.00002	0.00845	N/A	N/A
$s \in (0, 0.001]$	0.00024	0.00383	0.00905	0.24026
$s \in (0.001, 0.005]$	0.00004	0.00323	0.03563	0.27933
$s \in (0.005, 0.010]$	0.00125	0.00531	0.06200	0.26997
$s \in (0.010, 0.050]$	0.00165	0.00779	0.07517	0.26429

Table S10: Mean bias and RMSE in MAP estimates of the selection coefficient and dominance parameter across different ranges of the selection coefficient s with the parameters $\phi = 0.85$ and $\psi = 1$ (*i.e.*, scenario D in Table 1), corresponding to Figure S11. Mean bias and RMSE are calculated with 200 replicates for each case.

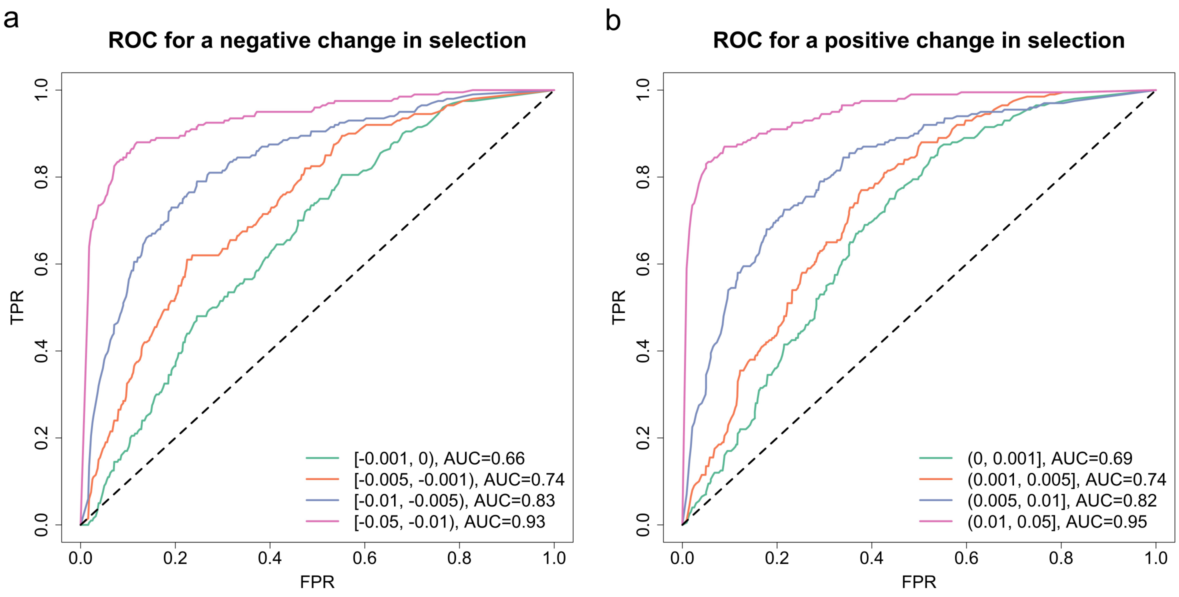

Figure S12: ROC curves for testing a change in selection across different ranges of the selection change Δs with the parameters $\phi = 0.85$ and $\psi = 1$ (*i.e.*, scenario D in Table 1. The AUC value for each curve is summarised. The simulated datasets for each range of the selection change are the same as those in Figure 6. ROC curves for (a) a negative change in selection and (b) a positive change in selection. Compared to Figure 6, the dominance parameter h is estimated rather than prespecified.

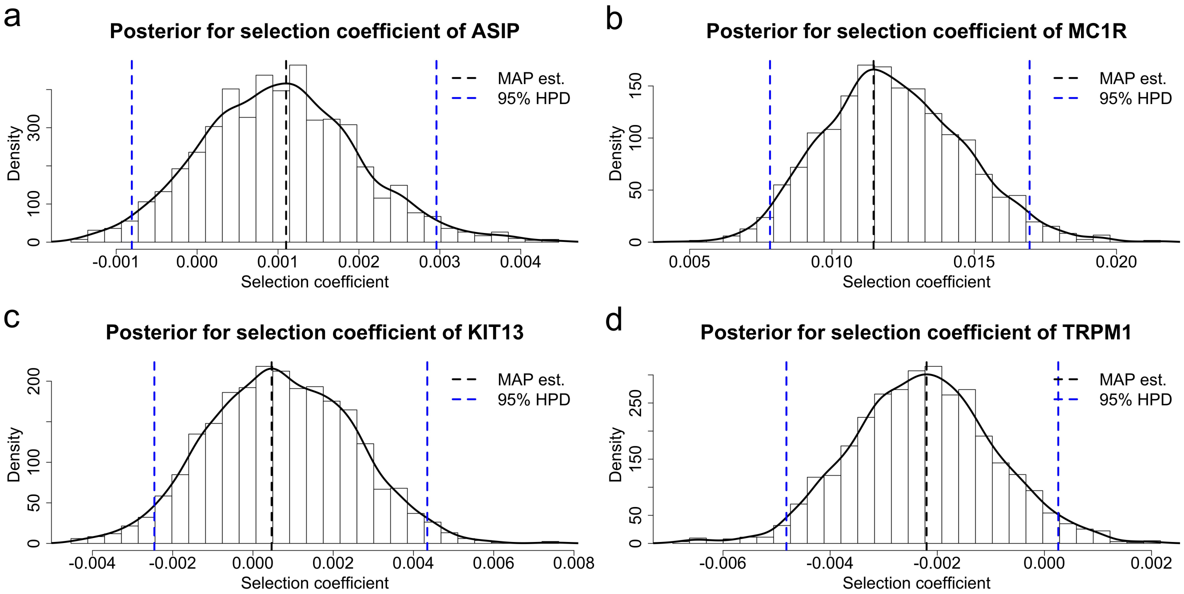

Figure S13: Posteriors for the selection coefficients of (a) *ASIP*, (b) *MC1R*, (c) *KIT13* and (d) *TRPM1*. We assume that the selection coefficient of each gene is fixed over time, and run our procedure on the ancient horse samples presented in Table S4 with the same sample exclusion criteria for each gene.

Gene	Sel. coeff.	MAP est.	95% HPD	Prob. for -ve.	Prob. for +ve.
ASIP	s	0.00110	$[-0.00081, 0.00296]$	0.1375	0.8625
MC1R	s	0.01146	$[\ 0.00782, 0.01695]$	0.0000	1.0000
KIT13	s	0.00047	$[-0.00246, 0.00434]$	0.3540	0.6460
TRPM1	s	-0.00220	$[-0.00482, 0.00026]$	0.9565	0.0435

Table S11: MAP estimates of the selection coefficients with their 95% HPD intervals, as well as posterior probabilities for negative selection and positive selection, for *ASIP*, *MC1R*, *KIT13* and *TRPM1*, corresponding to Figure S13. Prob. for -ve. and +ve. stand for posterior probabilities for negative selection and positive selection, respectively.

References

Bollback, J. P., York, T. L., & Nielsen, R. (2008). Estimation of $2N_e s$ from temporal allele frequency data. *Genetics*, *179*, 497–502.

Der Sarkissian, C., Ermini, L., Schubert, M., Yang, M. A., Librado, P. et al. (2015). Evolutionary genomics and conservation of the endangered Przewalski's horse. *Current Biology*, *25*, 2577–2583.

He, Z., Dai, X., Beaumont, M. A., & Yu, F. (2020a). Detecting and quantifying natural selection at two linked loci from time series data of allele frequencies with forward-in-time simulations. *Genetics*, *216*, 521–541.

He, Z., Dai, X., Beaumont, M. A., & Yu, F. (2020b). Estimation of natural selection and allele age from time series allele frequency data using a novel likelihood-based approach. *Genetics*, *216*, 463–480.

Ludwig, A., Pruvost, M., Reissmann, M., Benecke, N., Brockmann, G. A. et al. (2009). Coat color variation at the beginning of horse domestication. *Science*, *324*, 485–485.

Malaspinas, A.-S., Malaspinas, O., Evans, S. N., & Slatkin, M. (2012). Estimating allele age and selection coefficient from time-serial data. *Genetics*, *192*, 599–607.

Pruvost, M., Bellone, R., Benecke, N., Sandoval-Castellanos, E., Cieslak, M. et al. (2011). Genotypes of predomestic horses match phenotypes painted in Paleolithic works of cave art. *Proceedings of the National Academy of Sciences*, *108*, 18626–18630.

Schraiber, J. G., Evans, S. N., & Slatkin, M. (2016). Bayesian inference of natural selection from allele frequency time series. *Genetics*, *203*, 493–511.

Steinrücken, M., Bhaskar, A., & Song, Y. S. (2014). A novel spectral method for inferring general diploid selection from time series genetic data. *The Annals of Applied Statistics*, *8*, 2203–2222.

Wutke, S., Sandoval-Castellanos, E., Benecke, N., Döhle, H., Friederich, S. et al. (2018). Decline of genetic diversity in ancient domestic stallions in Europe. *Science Advances*, *4*, eaap9691.
